# Prognostic nomogram for the outcomes in acute stroke patients with intravenous thrombolysis

**DOI:** 10.3389/fnins.2022.1017883

**Published:** 2022-10-19

**Authors:** Zheng Ping, Li Min, Lu Qiuyun, Chen Xu, Bai Qingke

**Affiliations:** ^1^Key Laboratory and Neurosurgery, Shanghai Pudong New Area People’s Hospital, Shanghai, China; ^2^Department of Neurology, Shanghai Pudong New Area People’s Hospital, Shanghai, China; ^3^Department of Neurology, Shanghai Eighth People’s Hospital, Shanghai, China

**Keywords:** cerebral infarction, logistic regression model, ischemic stroke, OCSP, intravenous thrombolysis

## Abstract

**Background and purpose:**

The prediction of neurological outcomes in ischemic stroke patients is very useful in treatment choices, as well as in post-stroke management. This study is to develop a convenient nomogram for the bedside evaluation of stroke patients with intravenous thrombolysis.

**Materials and methods:**

We reviewed all enrolled stroke patients with intravenous thrombolysis retrospectively. Favorable outcome was defined as modified Rankin Score (mRs) less than 2 at 90 days post thrombolysis. We compared the clinical characteristics between patients with favorable outcome and poor outcome. Then, we applied logistic regression models and compared their predictability.

**Results:**

A total of 918 patients were enrolled in this study, 448 patients from one hospital were included to develop a nomogram, whereas 470 patients from the other hospital were used for the external validation. Associated risk factors were identified by multivariate logistic regression. The nomogram was validated by the area under the receiver operating characteristic curve (AUC). A nomogram was developed with baseline NIHSS, blood sugar, blood cholesterol level, part-and full anterior circulation infarction (OCSP type). The AUC was 0.767 (95% CI 0.653–0.772) and 0.836 (95% CI 0.697–0.847) in the derivation and external validation cohorts, respectively. The calibration plot for the probability of severe neurological outcome showed an optimal agreement between the prediction by nomogram and actual observation in both derivation and validation cohorts.

**Conclusion:**

A convenient outcome evaluation nomogram for patients with intravenous thrombolysis was developed, which could be used by physicians in making clinical decisions and predicting patients’ prognosis.

## Introduction

The prediction of neurological outcomes in ischemic stroke patients is very useful in treatment choices, as well as in post-stroke management (Jiang et al., 2021; Kerleroux et al., 2021). However, an object prediction system to evaluate the benefits of intravenous thrombolysis treatment in acute ischemic stroke patients is missing.

A nomogram is a scoring system based on a series of variables (Zhang et al., 2022b). Nomograms are extensively used in clinical sessions to predict the probability of an event. As nomograms have been extensively applied in oncology events such as: metastasis, survival status prediction, recurrence and response to treatment (Zhang et al., 2022d); cardiovascular disease (Wu et al., 2022) and brain vascular disease (Tang et al., 2022). This leads us to the application of a nomogram in the stroke field.

In this study, we aimed to create a nomogram for the baseline evaluation of patients with initial data on presentation to the emergency department. The neurologist might be able to use this simple tool to classify patients with intravenous thrombolysis due to poor prognosis immediately at presentation.

## Materials and methods

We enrolled 918 patients with ischemic stroke with thrombolytic therapy within 6 h of the stroke onset from July 2018 to June 2020 retrospective in two stroke centers in Shanghai, China. Neurological outcome was determined with modified Rankin Score (mRs) at about 90 days after the thrombolysis, and favorable outcome was defined as mRs score <2 (Wang et al., 2020). This study was approved by the Ethical Board of Shanghai Pudong New area People’s Hospital.

Stroke patients aged 18–80 years whose computed tomography (CT) scans showed no acute hemorrhage were recruited at local hospital consecutively. Patients with missing clinical data were excluded, and 488 patients with intact medical records were included as in-house dataset. Specifically, for stroke patients with an onset within 4.5 h, rtPA treatment should be delivered directly, while for patients whose onset was more than 4.5 h, rtPA should not be administered until the advanced magnetic resonance imaging (MRI) analysis is completed. These MRI sequences can be completed within 5–8 min. When the results from DWI and Flair are mismatched, the intravenous thrombolysis could be applied in patients with stroke onset > 4.5h, which is consistent with ESO guideline 2021.

Patients from our hospital (the primary cohort) were used for nomogram derivation, and those from the other hospital formed the external validation cohorts. Among the study population, 70% (*n* = 341) were randomly selected for the training set and the remaining 30% (*n* = 147) were assigned as the test set to prevent overfitting of the models. This was a retrospective observational cohort study; all data were analyzed anonymously and the informed consent of patients was waived. The baseline clinical data were collected when patients presented at the emergency Room before the thrombolysis.

Patients from other hospitals were used for external validation, including 316 stroke patients within 4.5 h of onset (control group) and 114 patients with wake-up stroke (WUS group).

### Statistical methods

Statistical analyses were conducted based on logistic regression to identify risk factors. Variables with a *p* value < 0.05 in univariate logistic regression were considered to be linked to the study outcomes and were further used for backward step-down logistic regression. The nomogram’s performance was measured by area under the receiver operating characteristic curve (AUC) and assessed by comparing nomogram-predicted versus observed incidences of the outcomes. External validation of the nomogram was conducted by calculating the total points of each patient in the validation cohort as per the established nomogram, followed by logistic regression in this cohort carried out by using the total points as a factor, and last, the AUC and calibration curve, bootstrap method and DCA were derived based on regression analysis. Two-tailed *p* < 0.05 was considered statistically significant. All the statistical analyses were conducted in R software V.4.1.2. R packages, namely “caret” for randomization of developing and validating groups (Kumar, 2018), “rms” for calibration plot, “pROC” to obtain the AUC (Sadatsafavi et al., 2022), “riskRegression” to perform Bootstrap method (Ozenne et al., 2017), and “rmda” to do the DCA (Brobbey et al., 2022), were used in the analysis.

## Results

A total of 500 patients were enrolled to the study. After excluding 12 patients with missing related clinical data, and 488 patients who underwent thrombolytic therapy were finally included as the in-house dataset. The mean age of the 488 patients was 59.44 years and 74.18% were men. Comparison of demographic variables between the high and low mRs groups are shown in the Table 1.

**TABLE 1:** Clinical data comparison in the in-house data.

Item	mRs_High	mRs_Low	Statistical value	*P* value
Gender (Male/Female)	275/87	99/27	NA	0.636
Age (years)	60.00 (52.00–67.00)	60.00 (53.25–67.00)	*t* = 1.243	0.447
First onset	13/361	2/112	NA	>0.9999
**Previous hypertension**	234/140	78/36	NA	0.304
**SysP (kPa)**	20.60 (18.67–22.50)	21.33 (19.50–22.67)	*t* = 1.432	**0.034**
DiaP (kPa)	12.00 (10.67–13.33)	12.00 (10.97–13.33)	*t* = 1.241	0.117
Previous DM	22/137	59/270	NA	0.8930
**DM_value (mmol/L)**	**5.50 (4.60–6.10)**	**5.20 (4.93–7.07)**	*t* = 2.928	**0.002**
Previous HTG	219/155	67/47	NA	1
**TG (mmol/L)**	1.54 (1.02–2.12)	1.40 (1.00–1.85)	*t* = 1.036	0.118
**TC (mmol/L)**	**4.72 (4.06–5.29)**	**4.34 (3.67–4.99)**	*t* = 3.821	**0.001**
**LDL (mmol/L)**	**2.90 (2.22–3.48)**	**2.60 (1.66–3.30)**	*t* = 3.095	**0.007**
Onset time (h)	1.96 (1.10–3.29)	2.00 (1.01–3.20)	*t* = 0.068	0.975
DNT (h)	1.41 (1.00–2.00)	1.33 (1.02–1.80)	*t* = 0.522	0.305
OTT (h)	3.50 (2.57–5.38)	3.58 (2.57–5.00)	*t* = 0.317	0.988
**NIHSS_Baseline**	**9.00 (6.25–13.75)**	**6.00 (5.00–9.00)**	*t* = 6.233	**<0.001**
**NIHSS_Day 7**	**8.00 (6.00–10.00)**	**2.00 (1.00–3.00)**	*t* = 9,565	**<0.001**

SysP, systolic pressure; DiaP, diastolic pressure; DM, diabetes; HTG, history of lipidemia; TG, triglyceride; TC, cholesterol; LDL, low-density lipoprotein; DNT, time from stroke to thrombolysis; OTT, Onset to therapy; NIHSS, National Institute of Health stroke scale. mRs ≥ 2, mRs high (unfavorable outcome); mRs < 2, mRs low (favorable outcome). The bold values indicate that there are statistical differences.

We found that in patients with higher mRs score at 90 days post onset, they have increased systolic pressure, blood glucose, TC, LDL and baseline NIHSS compared to those with lower mRs (BOLD with *p* value in Table 1), regardless of that patients with previous diabetes or hyperlipidemia. There is no difference in onset time, DNT, OTT, age, gender and rate of first onset between the two groups.

### Independent prognostic factors in the primary cohort

Unfavorable outcomes (mRs > 1) were observed in 114 (23.4%) of the 488 patients in the in-house dataset. Univariate logistic regression found that factors such as DM_value (OR = 1.17, 95% CI 1.04–1.31), NIHSS_Base (OR = 1.16, 95% CI 1.1–1.22), OCSP1 (OR = 2.08, 95% CI 1.08–3.99), OCSP2 (OR = 0.26, 95% CI 0.11–0.63), OCSP4 (OR = 6.34, 95% CI 1.81–22.28) and TC (OR = 1.36, 95% CI 1.09–1.7) levels were significantly linked to the severity of stroke. Multivariate analyses established that DM_value (adjusted OR (AOR) = 1.16, 95% CI 1.02–132), NIHSS_Base (AOR = 1.13, 95% CI 1.06–1.2), OCSP1 (AOR = 4.16, 95% CI 1.69–10.28), OCSP4 (AOR = 6.34, 95% CI 1.81–22.28) and TC (AOR = 1.38, 95% CI 1.08–1.75) levels were independent risk factors for severe neurological outcome (Table 2).

**TABLE 2:** Logistic regression in the primary cohort for stroke patients.

	Univariate logistic			Multivariate logistic		
Parameter	OR.x	CI.x	P.x	OR.y	CI.y	P.y
Age	1.02	0.99–1.04	0.232			
DiaP	1.08	0.93–1.25	0.335			
DM	0.75	0.36–1.57	0.442			
DM_value	1.17	1.04–1.31	**0.007**	1.16	1.02–1.32	**0.02**
DNT	1.1	0.84–1.44	0.468			
First onset	1.35	0.29–6.39	0.702			
Gender	0.92	0.51–1.65	0.773			
HBP	1.63	0.95–2.79	0.077			
HTG	0.89	0.53–1.48	0.649			
LDL	1.16	0.96–1.41	0.129			
NIHSS_Base	1.16	1.1–1.22	**0**	1.13	1.06–1.2	**0**
OCSP1	2.08	1.08–3.99	**0.028**	4.16	1.69–10.28	**0.002**
OCSP2	0.26	0.11–0.63	**0.003**			
OCSP3	0	0-Inf	0.984			
OCSP4	6.34	1.81–22.28	**0.004**	11.28	2.17–58.51	**0.004**
Onset_Time	1.02	0.89–1.15	0.816			
OTT	1.01	0.92–1.1	0.888			
SysP	1.09	1–1.2	0.057			
TC	1.36	1.09–1.7	**0.006**	1.38	1.08–1.75	**0.009**
TG	0.85	0.65–1.11	0.238			
TOAST1	1.35	0.26–7.1	0.723			
TOAST2	0.65	0.22–1.97	0.45			
TOAST3	1.01	0.44–2.32	0.987			
TOAST4	3.42	0.47–24.63	0.223			

HBP, History of Hypertension; OCSP1, Part anterior circulation infarction; OCSP2, Post circulation infarction; OCSP3, Lacunar infarction; OCSP4, Full anterior circulation infarction; TOAST1, Cardio embolism; TOAST2, Small-artery occlusion Lacunar; TOAST3, Large-artery atherosclerosis; TOAST4, acute stroke of other determined etiology or stroke of other undetermined etiology. The bold values indicate that there are statistical differences.

**FIGURE 1:**
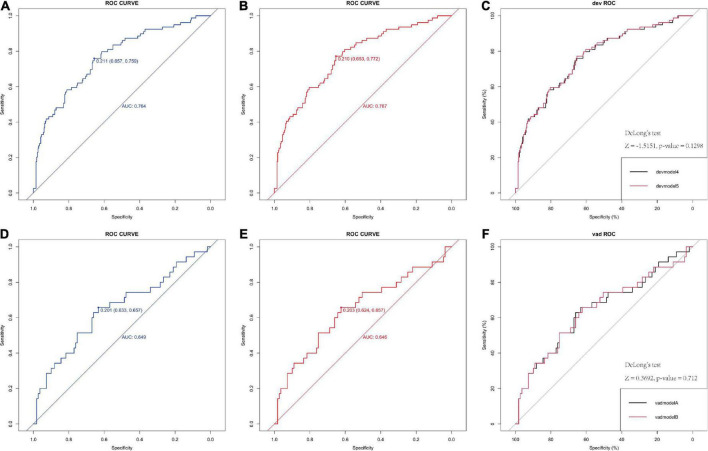
Receiver operating characteristic (ROC) of the logistic model in predicting poor neurological outcome in derivation and validation cohorts. **(A,B)** ROC curve of 6-factor and 5-factor logistic model for the developing in-house data which show the AUC value. **(C)** The comparison between the two models by the Delong’s test. **(D,E)** ROC curve of 6-factor and 5-factor logistic mode for the validating in-house data which show the AUC value, indicating both models have good predict ability. **(F)** The comparison between the two models by the Delong’s test. AUC, area under the receiver operating characteristic curve.

The logistic model indicated good discriminative ability with an AUC value of 0.767 in the development model and 0.646 in the internal validation model (95% CI 0.624–0.657) (Figure 1). The factor OCSP2 was not included in the final model (devmodel 4), as its *p* value (*p* = 0.1298) by Delong’s test was insignificant, which is the same case as the internal validation group (*p* = 0.712).

### Validation of predictive accuracy of the logistic model for outcome severity

**FIGURE 2:**
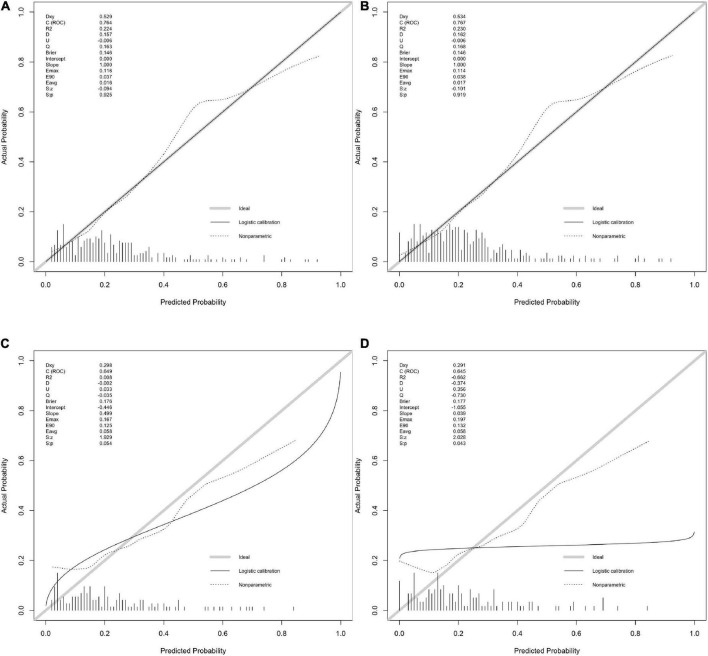
The calibration with rms package. **(A,B)** Calibration plot for 6-factor and 5-factor logistic model for the developing in-house data which show the c(ROC) and S.p value. **(C,D)** Calibration plot for 6-factor and 5-factor logistic model for the validating in-house data which show the c(ROC) and S.p value.

The calibration plot for the probability of poor neurological outcome indicated an optimal agreement between the predictions through the 6-factor logistic model (added with OCSP2, Figure 2A) and 5-factor logistic model (Figure 2B).

The calibration curve also indicated a good agreement between 5-factor logistic model (Figure 2C) and 4-factor logistic model (Figure 2D) in the internal validation model with a similar ROC value.

**FIGURE 3:**
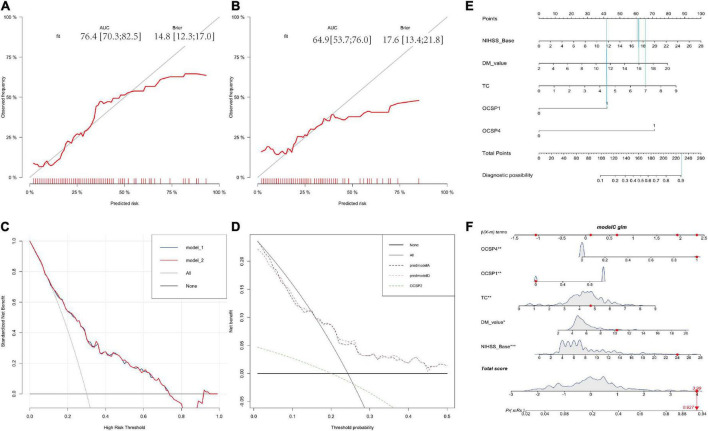
Calibration and DCA validation for the logistic models for the in-house dataset. **(A,B)** Calibration plot for the developing and validating the in-house data which show the AUC and Brier value. **(C,D)** The DCA validation for the developing and validating the in-house data which show both models have good net benefits. **(E)** The nomogram for distinguishing risk factors. **(F)** Prognostic GLM for stroke patients with intravenous thrombolysis. Model 1 is the 6-factor logistic model (added with OCSP2) and Model 2 is the 5-factor logistic model in the developing in-house dataset; Premodel A is the 6-factor logistic model (added with OCSP2) and Premodel B is the 5-factor logistic model in the validating in-house dataset.

A correlation matrix was verified by the Bootstrap method. The formula was formed with 5 factors first and calibration score was calculated and the calibration figure was plotted in Figures 3A,B for the developed and internal validated data, which showed that the AUC value is 76.4 (95%CI: 70.3–82.5) and 64.9 (95%CI: 53.7–76.0), respectively. The Brier score was 14.6 and 17.6, while both were less than 25. We further used DCA method to verify the models. We found both models in the training data (Figure 3C) and both models in the internal validated data (Figure 3D) have good net benefits. When we added the OCSP2 in the internal dataset for the DCA analysis, it demonstrated a poor net benefit compared to the logistic models.

**TABLE 3:** Clinical characteristics of patients with different datasets.

Parameters	Level	In-house	External I	External II	*P* value^#^
*n*	NA	488	316	114	NA
Age	NA	60.00 (52.00–67.00)	60.0(53.0–68.0)	62.0(53.2–67.0)	0.3428
Gender	Male	362 (74.18%)	205 (64.9%)	75 (65.8%)	0.1113
	Female	126 (25.82%)	111 (35.1%)	39 (34.2%)	
HBP	With	312 (63.93)	206 (65.2%)	77 (67.5%)	0.7567
	Without	176 (36.07%)	110 (34.8%)	37 (32.5%)	
SynP	NA	20.8 (18.67–22.53)	20.7 (18.7–22.7)	21.5 (20.0–22.7)	0.2628
DiaP	NA	12.00 (10.67–13.33)	12.0 (10.7–13.3)	12.0 (11.3–13.3)	0.3683
DM	With	81 (16.6%)	60 (19.0%)	16 (14.0%)	0.4413
	Without	407 (83.40%)	256 (81.0%)	98 (86.0%)	
DM_value	NA	5.30 (4.70–6.30)	5.3 (4.7–6.3)	5.4 (4.9–6.4)	0.6682
HTG	With	286 (58.61%)	172(54.4%)	80 (70.2%)	0.1380
	Without	202 (41.39%)	144 (45.6%)	34 (29.8%)	
TG	NA	1.50 (1.02–2.10)	1.4 (1.0–2.1)	1.6 (1.2–2.2)	0.2650
TC	NA	4.43 (3.78–5.08)	4.5 (3.9–5.1)	4.6 (3.9–5.1)	0.3204
LDL	NA	2.70 (1.73–3.30)	3.0 (2.4–3.5)	3.1 (2.4–3.5)	<0.0001*
TOAST	1	430 (88.11%)	161 (50.9%)	55 (48.2%)	<0.0001*
	2	40 (8.20%)	113 (35.8%)	38 (33.3%)	
	3	14 (2.87%)	33 (10.4%)	19 (16.7%)	
	4	4 (0.82%)	9 (2.8%)	2 (1.8%)	
OCSP	1	353 (72.34%)	187 (59.2%)	68 (59.6%)	0.0002*
	2	135 (27.68%)	129 (40.8%)	46 (40.4%)	
NIHSS_Baseline	NA	7.00 (5.00–10.00)	7.0 (6.0–10.0)	8.0 (6.0–10.8)	0.3910
NIHSS_Day7	NA	2.00 (1.00–5.00)	2.0 (1.0–4.0)	2.0 (1.0–5.0)	0.3753

*No difference between the External I and External II; ^#^Comparison among the three groups.

**TABLE 4:** Confusion matrix for the in-house training dataset.

Confusion matrix	True prediction	False prediction
True value	258	59
False value	7	20

The mis-class error is 0.1919. *n* = 344 in the in-house training dataset.

**TABLE 5:** Confusion matrix for the in-house validation dataset.

Confusion matrix	True prediction	False prediction
True value	104	28
False value	5	7

The mis-class error is 0.2292. *n* = 144 in the in-house validation dataset.

### Risk prediction nomogram for outcome severity

The nomogram for outcome prediction of stroke patients was developed in accordance with the multivariate regression model (5 factors), suggesting that these five parameters are all independent risk factors for the neurological outcome with all ORs > 1 indicating that all of these factors are risk factors (Figures 3E,F). For example, if a patient presented with part-anterior circulation infarction (OCSP1 = 42 points), initial NIHSS = 17 (60 points), blood glucose = 16 (61 points), TC level is 7 (66 points). The total is 229 points, which indicated that the probability of this patient to develop a poor neurological outcome is a bit higher than 90% (Figure 3E).

### External validation

To validate the prevalence and efficiency of this logistical model in stroke after intravenous thrombolysis, we verified this in other two external datasets including 316 stroke patients within 4.5 h of onset (control group) and 114 patients with wake-up stroke (WUS group). Unfavorable outcomes (mRs > 1) were observed in 85 (26.9%) of the 316 and 38 (33.3%) of the 114 patients in the two out-house dataset. Both groups of patients were treated with intravenous rt-PA. There was no significant difference in NIHSS scores and 90-day mRs between the two groups (Table 3), which indicated that these two datasets could be used for external validation independently. The clinical characteristics of patients from different datasets were compared in Table 3. We further explored the confusion matrix for the training set and validation set, respectively and summarized in Tables 4, 5. The mis-class error for both is 19.19 and 22.92%.

**FIGURE 4:**
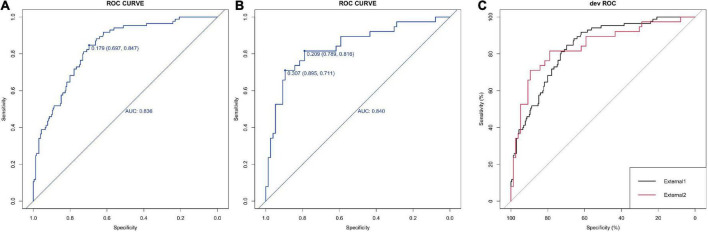
Calibration and DCA validation for the logistic models for the external datasets. **(A,B)** ROC curve of the 5-factor logistic model for two external datasets which show the AUC value. **(C)** The comparison between the ROC curve by the Delong’s test.

**FIGURE 5:**
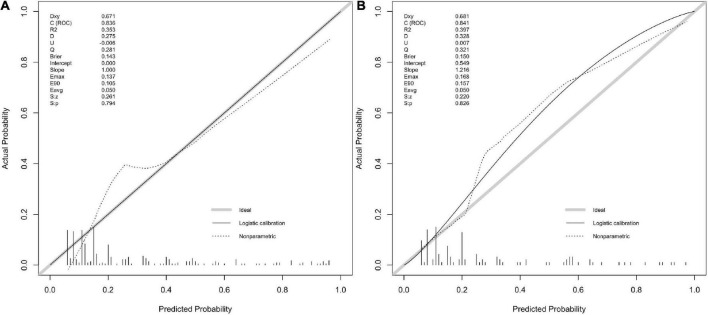
**(A,B)** Calibration plot for the 5-factor logistic model for two external datasets which show the c(ROC) and S.p value.

For the logistic regression model, the predictive power of the was 0.836 (95%CI: 0.697–0.847) and 0.840 (95%CI:0.711–0.895), respectively (Figures 4A,B). No significant difference in AUC values between External 1 and External 2 cohorts (*p* = 0.497) was found by the delong’s test (Figure 4C). Again, the models were further validated with calibration showing the S.p was 0.794 and 0.826 (Figures 5A,B).

**FIGURE 6:**
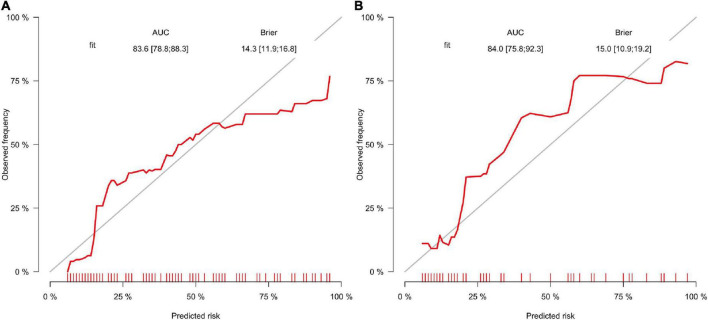
**(A,B)** Calibration plot for two external datasets which show the AUC and Brier value.

Again, the Bootstrap method was applied to assess the correlation matrix and it was found that the AUC value is 83.6 (95%CI: 78.8–88.3) and 84.0 (95%CI: 75.8–92.3), respectively. The Brier score was 14.3 and 15.0, while both were less than 25 (Figures 6A,B).

**FIGURE 7:**
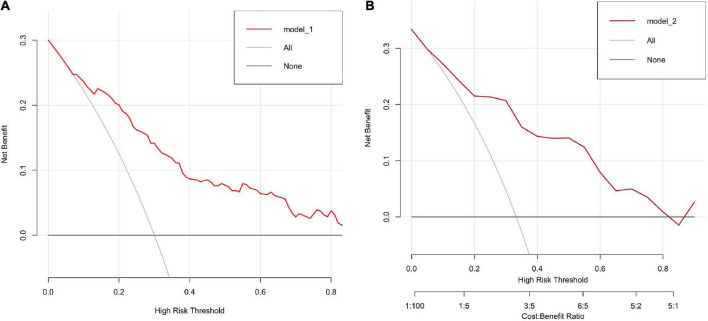
**(A,B)** The DCA validation for two external datasets which show both datasets have good net benefits.

We further verified the external datasets with DCA method as well. We found both models in External 1 (Figure 7A) and External 2 (Figure 7B) have good net benefits. In addition, the confusion matrix for the External 1 and 2 set was summarized in Tables 6, 7. The mis-class error for both is 20.89% and 19.30%.

**TABLE 6:** Confusion matrix for the External I dataset.

Confusion matrix	True prediction	False prediction
True value	217	52
False value	14	33

The mis-class error is 0.2089. *n* = 316 in the External I dataset.

**TABLE 7:** Confusion matrix for the External II dataset.

Confusion matrix	True prediction	False prediction
True value	72	18
False value	4	20

The mis-class error is 0.1930. *n* = 114 in the External II dataset.

## Discussion

Our study demonstrated that the use of logistic models can accurately predict neurological outcomes in acute stroke patients with intravenous thrombolysis. First, we show that stroke patients with higher NIHSS score on day7 have increased systolic pressure, blood glucose, TG, TC, LDL levels and baseline NIHSS compared to those with lower NIHSS score, while no difference of distribution in patients with previous diabetes or hyperlipidemia. This suggests that it is critical to control the blood pressure in acute stroke patients (Zhang et al., 2019, 2022c; Chen et al., 2021; Mazighi et al., 2021), especially decreasing blood glucose and lipid level including TG, TC and LDL, regardless of patients with previous history or not.

As the 7-day NIHSS is an early assessment score in stroke patients and it might be not available for some patients (Mistry et al., 2021). Therefore, we did not include it in the logistic model, instead of the baseline NIHSS. According to the clinical features and laboratory findings, we established and validated prognostic nomogram for neurological outcome of stroke patients. The proposed nomogram revealed excellent discrimination in both the training and testing cohorts. In addition, accurate predictions for outcome severity using the developed nomograms were indicated by calibration curves, bootstrap and DCA methods.

In this study, multivariate logistic regression was used for predicting a binary neurological outcome based on the mRs at 3 months post stroke. This nomogram was developed based on five predictors, comprising baseline NIHSS, blood sugar level, blood cholesterol and OCSP types: part and full anterior circulation infarction. These variables were established to be linked with a poor prognosis of stroke patients with intravenous thrombolysis in certain earlier studies. Multivariate analysis showed that the OCSP with part or full anterior circulation infarction is also a risk factor with a higher OR value, indicated that patients with the anterior circulation infarction are more likely to develop poor neurological outcome after the thrombolysis. Meanwhile, we found both TC and blood sugar levels had a statistically significant association with neurological outcome, regardless of patients with previous history of DM or hypertension. Metabolic syndrome is a risk factor for the poor outcome in stroke (Xu et al., 2022). After reducing the risk factors, most stroke patients might have a better prognosis after thrombolysis, while some of them might be not. Only targeting blood lipids or blood sugar may overestimate the risk of stroke, and the combination of these five indicators in our nomogram may overcome this disadvantage. These variables were objective, easy to acquire and quickly assessable. By combining these non-specific variables, good discrimination in both cohorts was obtained with this nomogram.

We calculated predicted risk and relative scores in the nomogram. As per the nomogram, patients with a >2 points (Figure 3E) score would have a > 60% chance of developing severe prognosis of stroke, a patient with a score of > 4 would develop a severe case with a probability of 90%. A nomogram is beneficial to emergency neurologist for evaluating patients immediately, especially in rural areas, where the MRI is not available. This predictive nomogram may be used in optimally estimating individualized disease-related risks that simplify patient management-related decision-making.

Importantly, we verified this model with both internal validated dataset and external datasets from other hospitals. The calibration, bootstrap and DCA results were quite consistent in both internal and external validated datasets with the ROC value between 0.65 and 0.75 and the Brier score is both less than 25. For the DCA validation results, the models with five factors obtained relatively good net benefits in all datasets.

Therefore, our study established a nomogram to be used as a support tool for predicting stroke cases with thrombolysis. Chen group also used nomogram to assess the outcome of patients with ischemic stroke after intravenous thrombolysis and found the accuracy of the nomogram is 0.641 and 0.627 in the training cohort and validation cohort (Hu et al., 2022). They suggested that the accuracy of the nomogram needs to be improved. A recent study led by Wang yongjun group identified serum glucose level is an independent risk factor in the poor 3-month functional recovery in stroke patients with the univariate and multivariate logistic regression study (Che et al., 2022). Mehta et al. also used the logistic regression study to find DM, dyslipidemia, baseline NIHSS, random blood sugar, dense cerebral artery sign, age and glucose level on admission were predicting factors in poor outcome (Mehta et al., 2017). One group found the nomogram including initial NIHSS, delta NIHSS, hypertension, Hhcy, HDL-C/LDL-C hold a very high AUC-ROC value of the training cohort with 0.872 and 0.900 in the test cohort, which is higher than the predictive ability of our model. In their study, the authors included several novel blood markers such as: Hhcy, HDL-C/LDL-C, which indicates that these lipid markers might be very important in the pathology of stroke (Lv et al., 2020). Similar results were obtained from Huan Tang et al., and the 3-month poor outcome is related to the baseline elevated SBP, baseline NIHSS, prior hyperlipemia, cardioembolic stroke (Tang et al., 2021). Few studies correlated the neurological outcome with logistic regression model rather than the nomogram (Çetiner et al., 2018; Tork et al., 2020; Cui et al., 2022), and most of them were focusing on the complications in stroke patients after thrombolysis. Manuel et al. reported that SICH nomogram is able to predict the symptomatic intracerebral hemorrhage after intravenous thrombolysis for stroke with a ROC at 0.739 (Cappellari et al., 2018). Weng et al. also reported that AUC-ROC values of the nomogram regarding the risk of intracranial hemorrhage is 0.887 and 0.776 in the training and testing sets (Weng et al., 2022). Zhou et al. obtained the similar results with AUC-ROC values of the nomogram are 0.828 and 0.801, respectively (Zhou et al., 2020). Two other studies applied the nomogram to investigate the risk of hemorrhagic transformation for acute ischemic stroke with AUC value at 0.889 (Zhang et al., 2022a) and 0.9562 (Wu et al., 2020). These findings investigated the clinical characteristics of stroke patients have a predictable ability in hemorrhagic complications, while they did not further investigate the neurological outcome at the chronic stage such as 3 months post onset. Nonetheless, certain limitations of this study need mentioning. First, due to this study’s retrospective design, some valuable variables such as symptoms and certain imaging scores (CT features and MRI parameters) were inaccessible. Variables used as inputs to the logistic algorithms were those that are traditionally attainable or evaluated in most cases. However, the prediction might be influenced slightly according to the variables and may be adjusted with consideration for their availability when incorporating data from MRI imaging. Patients with intact imaging information are required to be included to perform the radiomics (Miao et al., 2022). Thus, the discrimination of this nomogram and other risk scoring systems was not compared. Second, this study enrolled only two independent hospitals. Both hospitals were located in Shanghai; thus, other validation cohorts from other cities, even other countries would encourage widespread application of this nomogram.

## Conclusion

A five-variable risk prediction nomogram was developed based on demographic and routine laboratory tests, which accurately predicts the probability of neurological outcome. This nomogram can be used to help risk stratify stroke patients with thrombolysis on presentation to the emergency department and provide useful strategy in improving the outcome.

## Data availability statement

The original contributions presented in this study are included in the article/Supplementary material, further inquiries can be directed to the corresponding author.

## Ethics statement

The studies involving human participants were reviewed and approved by the Ethical Board of Shanghai Pudong New Area People’s Hospital. Written informed consent for participation was not required for this study in accordance with the national legislation and the institutional requirements.

## Author contributions

ZP and BQ designed the clinical data and did the bioinformatical analysis with LM. LQ collected the clinical data. ZP and CX wrote the draft. All authors approved the submitted version.
