# Supplementary material for: Prognostic nomogram for the outcomes in acute stroke patients with intravenous thrombolysis
Source: Front Neurosci. 2022 Oct 19;16:1017883. doi: 10.3389/fnins.2022.1017883 (PMC9627298; doi:10.3389/fnins.2022.1017883)
Supplement: Supplementary file 1 [file Data_Sheet_1.PDF]

ID	G	A	F	HB	SP	DP	DM	DM2	HTG	G
**401**	2	65	2	2	17.33333	10.66667	2	20.6	2	1.69
**362**	1	63	2	2	26.66667	13.33333	1	19.9	2	1.75
**140**	1	48	2	1	20	10.66667	1	16.2	1	0.83
**123**	1	58	2	2	23.2	12.53333	2	15.3	1	1.25
**75**	2	69	2	2	24	10.26667	2	14.1	1	1.16
**143**	2	54	2	2	20	12	1	14.1	1	2.51
**351**	2	45	2	2	21.33333	13.33333	1	13.8	2	4.43
**453**	1	43	2	2	22.13333	13.06667	2	13.6	2	7.23
**205**	1	78	2	2	22.66667	12.8	2	13.4	2	1.79
**407**	1	63	2	2	22.13333	11.86667	2	13.1	1	2.2
**35**	1	62	2	2	21.33333	12	2	12.8	1	2.8
**94**	2	67	2	1	22.66667	12.8	2	12.8	1	3.09
**13**	2	51	2	2	21.2	10.66667	2	12.8	2	1.47
**247**	2	68	2	2	20.26667	10.66667	1	12	1	0.85
**338**	1	64	2	2	26.66667	13.33333	2	11.8	1	1.94
**475**	2	71	2	2	22.66667	10.66667	1	11.7	2	2.23
**365**	1	51	2	2	20.53333	12	1	11.5	1	2.01
**253**	1	54	2	2	21.6	13.86667	2	11.5	2	5.84
**26**	2	74	2	2	18.66667	9.333333	2	11	2	3.32
**423**	2	62	2	2	20.26667	12.8	2	10.8	1	1.5
**467**	1	62	2	2	20.93333	10.93333	1	10.6	1	0.74
**468**	1	47	2	2	19.6	12.66667	1	10.6	2	2.55
**298**	1	46	2	2	20.4	14	2	10.6	2	5
**163**	1	65	2	2	18	10.66667	2	10.4	2	1.03
**3**	1	55	2	2	22	14	1	10.3	1	1.49
**214**	1	66	2	2	22.66667	12.53333	2	10.3	2	1.53
**346**	2	66	2	2	24	12	2	10	1	1.98
**300**	1	72	2	2	24	12	2	9.9	2	0.89
**360**	2	75	2	2	22.13333	11.86667	2	9.7	1	1.59
**57**	1	74	2	2	22.66667	12	1	9.5	1	0.75
**18**	2	75	2	2	21.33333	12	1	9.5	2	0.87
**392**	1	71	2	1	18.66667	10.66667	2	9.5	2	1.35
**399**	1	47	2	2	21.06667	13.06667	1	9.5	2	12.95
**424**	2	56	2	1	18.66667	10.4	2	9.4	2	0.78
**405**	1	55	2	2	22	14.4	2	9.2	1	0.97
**175**	1	56	2	2	24	13.33333	2	9.2	1	1.1
**146**	1	64	2	1	20.26667	12	2	9.1	2	7.15
**193**	1	76	2	2	26.4	11.46667	2	8.95	1	1.59
**12**	1	43	2	2	20	14.53333	1	8.8	1	1.72
**380**	1	59	1	2	22.53333	12.66667	2	8.7	1	1.74
**455**	1	37	2	1	15.33333	9.333333	1	8.7	1	1.44
**153**	1	55	2	2	21.2	12.4	2	8.6	2	2.54
**194**	1	47	2	2	19.6	10.66667	1	8.5	1	2.89
**46**	1	65	2	2	28.4	10.8	1	8.5	1	1.42
**293**	1	56	2	2	22.66667	14.26667	2	8.3	1	1.37
**329**	1	56	2	1	18.66667	10.66667	2	8.3	2	3.01
**17**	1	53	2	1	17.33333	12	1	8.2	1	1.43

132	1	69	2	2	21.33333	12	2	8.2	1	1.33
166	2	77	2	1	20	12	1	8.1	2	1.92
283	1	66	2	2	18.66667	12	2	8.1	1	1.35
235	1	63	2	2	21.33333	13.06667	2	8.1	2	2.54
339	1	55	2	2	21.33333	15.2	1	8	1	2.78
118	1	64	2	2	26.66667	14.66667	2	8	2	0.89
227	1	53	2	2	21.33333	14.66667	1	8	1	1.98
186	1	55	2	1	24.13333	13.06667	1	8	2	2.97
255	2	50	2	2	33.33333	17.33333	1	8	1	3.64
275	1	58	2	1	22.4	11.73333	1	7.9	2	1.35
234	1	66	2	2	17.86667	10.66667	2	7.9	1	1.33
386	2	53	2	2	22	12	2	7.9	2	1.75
10	1	76	2	2	18.4	9.6	1	7.8	1	0.72
347	1	56	2	1	16	12	1	7.8	2	3.91
98	2	57	2	2	22.13333	13.2	1	7.8	1	0.69
445	1	59	2	2	18.66667	12	2	7.8	1	2.21
150	1	62	2	2	21.33333	14.66667	1	7.7	1	1.69
326	1	65	2	2	17.6	9.333333	1	7.7	1	1.29
9	1	45	2	2	18.26667	10.8	1	7.6	1	0.73
156	1	69	2	2	22.66667	13.33333	1	7.6	1	1.32
334	1	45	2	2	21.33333	13.33333	1	7.6	1	3.61
22	2	36	2	2	17.33333	12	1	7.5	2	2.29
8	2	75	2	1	22.53333	8	1	7.5	1	1.11
99	2	65	1	2	23.33333	11.46667	1	7.4	1	2.25
167	1	71	2	1	20	9.333333	1	7.3	2	0.96
141	1	79	1	2	20	13.33333	1	7.3	2	1.05
144	2	76	2	2	22.66667	12	2	7.3	2	1.68
428	1	52	2	2	22.26667	10.66667	1	7.3	1	5.49
87	2	51	2	1	21.6	13.73333	2	7.26	1	0.34
353	2	66	2	2	19.2	12.8	2	7.2	1	1.62
76	1	75	2	2	23.46667	12	1	7.1	1	1.37
147	1	72	2	2	20.8	13.73333	2	7.1	2	0.83
240	1	52	2	1	18.66667	10.66667	1	7.1	1	1.37
324	1	66	2	2	18.4	11.33333	1	7.1	2	1.88
21	2	47	2	1	22.4	14.4	1	7	1	1.53
168	1	60	2	1	18.66667	12	2	7	2	0.71
404	1	56	1	2	17.33333	9.333333	1	7	1	1.75
122	2	68	2	1	18.66667	10.66667	1	7	2	0.73
27	1	75	2	1	24.13333	13.06667	1	7	1	4.49
245	1	74	2	2	21.33333	12	1	7	1	1.42
460	1	62	2	2	24.13333	13.2	2	7	2	1.53
92	1	76	2	2	25.33333	14.66667	1	6.9	2	1.15
230	2	75	2	2	20.53333	10.13333	2	6.9	2	2.09
199	1	66	2	2	23.73333	11.73333	2	6.8	2	2.31
62	1	47	2	1	20	13.33333	1	6.8	1	0.93
292	1	56	2	2	20	12	1	6.8	1	1.69
164	1	54	1	2	18.93333	13.73333	1	6.7	1	0.79
177	1	59	2	2	18.4	10.66667	1	6.7	1	4.53

**151**	2	61	1	2	23.6	10.66667	1	6.7	1	2.23
**238**	1	57	2	2	23.06667	12.93333	1	6.7	1	1.63
**183**	2	69	2	2	20.66667	9.333333	1	6.7	2	0.77
**4**	1	46	2	2	18.4	11.33333	1	6.6	1	1.42
**69**	1	50	2	2	22	14.66667	1	6.6	1	1.8
**181**	2	56	2	2	21.33333	12	2	6.6	2	0.73
**323**	1	68	2	2	22.13333	9.333333	1	6.6	1	0.84
**45**	1	52	2	2	21.33333	16	1	6.5	2	5.19
**120**	2	52	2	1	22.66667	14.66667	1	6.5	1	1.09
**127**	2	67	2	2	23.73333	12	2	6.5	2	0.91
**74**	1	40	2	1	18.66667	12	1	6.5	2	0.89
**304**	1	65	2	2	20.8	13.33333	1	6.5	2	2.09
**115**	1	63	2	1	18.66667	12	1	6.4	2	1.01
**11**	1	59	2	1	15.73333	10.8	1	6.4	2	2.83
**61**	1	62	2	2	23.46667	13.6	1	6.4	1	1.54
**213**	2	70	2	2	22.66667	11.2	1	6.4	2	2.18
**64**	1	69	2	2	20	12	1	6.4	1	1.34
**125**	2	66	2	2	18.13333	10.66667	2	6.4	2	1.3
**229**	1	76	2	2	21.6	12	1	6.4	1	2.34
**430**	1	50	1	2	24	14.66667	2	6.4	1	1.84
**109**	1	78	2	2	21.33333	12	1	6.4	1	4.53
**464**	1	33	2	1	20.93333	13.6	1	6.4	1	2.75
**420**	1	65	2	2	17.33333	10.66667	2	6.32	1	0.88
**474**	1	65	2	2	25.33333	13.33333	2	6.3	2	2.38
**232**	2	70	2	2	18	10	1	6.3	1	0.94
**418**	1	67	2	2	22.4	11.33333	1	6.3	2	1.4
**422**	1	64	2	2	17.33333	12	2	6.3	1	1.07
**385**	1	56	2	2	20.53333	13.86667	1	6.3	2	2.07
**483**	1	73	2	1	17.33333	10.66667	2	6.3	1	1.26
**261**	2	46	2	2	16	9.333333	1	6.3	1	5.2
**457**	1	80	2	2	18.4	10.53333	2	6.3	1	1.81
**161**	1	53	2	2	25.33333	14.66667	1	6.2	2	1.79
**269**	2	67	2	1	22.8	13.06667	1	6.2	1	0.89
**208**	1	71	2	2	23.2	11.2	2	6.2	1	1.72
**377**	1	68	2	2	18	12	1	6.2	1	3.75
**136**	2	51	2	1	26.66667	16	1	6.2	1	0.6
**330**	1	51	2	2	21.33333	14.66667	1	6.2	2	6.97
**266**	2	42	2	2	26.66667	13.33333	1	6.1	2	1.44
**343**	2	69	2	2	21.33333	13.33333	1	6.1	1	0.88
**121**	2	42	2	1	14.93333	2.666667	1	6.1	2	1.09
**79**	2	66	2	2	17.6	9.866667	2	6.1	1	1.01
**66**	1	72	2	2	24	14.66667	1	6.1	1	1.72
**72**	1	44	2	2	21.33333	14.66667	1	6.1	2	2.12
**32**	2	72	2	2	22.66667	13.33333	2	6.1	2	4.87
**111**	1	47	2	2	22.93333	12.66667	1	6.1	1	4.49
**248**	1	68	1	1	22.53333	9.333333	1	6.1	1	1.35
**384**	1	57	2	2	23.6	13.06667	1	6.1	1	1.16
**441**	2	64	2	2	21.86667	11.06667	1	6.1	2	1.46

**33**	1	67	2	1	17.33333	9.333333	1	6.03	1	1.2
**5**	1	52	2	1	19.46667	11.73333	1	6	1	0.47
**223**	2	66	2	1	23.46667	12.8	1	6	1	1.87
**281**	1	69	2	2	23.6	14.4	2	6	2	1.01
**282**	1	67	2	1	18.66667	12	1	6	2	2.26
**284**	1	63	2	2	21.46667	12.93333	1	6	2	1.64
**228**	1	52	2	1	19.33333	14.66667	1	6	1	1.36
**85**	2	74	2	2	20	12	1	6	2	2.19
**369**	1	77	2	2	20.8	10.66667	1	6	1	3.3
**373**	1	72	2	2	17.33333	12	1	6	1	1.63
**452**	2	47	2	1	19.33333	12.4	1	6	1	0.81
**190**	1	63	2	2	25.73333	13.46667	1	6	2	1.95
**1**	2	78	2	2	22.66667	9.333333	1	5.9	2	1.28
**425**	1	56	2	2	24	14.66667	1	5.9	2	2.68
**382**	1	58	2	2	23.73333	13.06667	1	5.9	2	2.1
**333**	1	46	2	2	23.2	13.33333	1	5.9	1	1.65
**393**	1	64	2	1	19.06667	11.2	1	5.9	2	2.6
**30**	2	71	2	2	24.26667	11.6	2	5.8	1	2.16
**204**	1	51	2	2	22.4	12.4	2	5.8	2	0.39
**212**	1	56	2	1	21.73333	12.26667	1	5.8	2	3.46
**274**	1	67	2	1	21.86667	10	1	5.8	2	3.86
**349**	2	51	2	2	20	12	1	5.8	2	1.87
**100**	1	42	2	1	18.66667	10.66667	1	5.8	1	0.58
**358**	1	52	2	2	23.86667	13.73333	1	5.8	1	3.53
**67**	2	61	2	2	21.06667	12.53333	1	5.8	1	0.9
**130**	1	60	2	1	21.33333	12	1	5.8	2	2.08
**157**	1	65	2	2	21.33333	12	2	5.8	2	1.81
**311**	1	60	2	1	21.86667	12	1	5.8	1	1.05
**314**	2	54	2	2	20.13333	11.2	1	5.8	1	0.9
**463**	1	55	2	2	20	12	2	5.8	2	0.7
**28**	1	59	2	2	22.13333	10.66667	1	5.7	1	1.4
**40**	1	62	2	1	21.06667	11.73333	1	5.7	1	1.08
**271**	1	44	2	1	23.46667	14.13333	1	5.7	2	1.67
**104**	2	58	2	1	17.33333	9.333333	1	5.7	2	1.49
**154**	1	78	2	2	21.33333	10.66667	2	5.7	2	1
**309**	2	60	2	1	18.66667	12	1	5.7	1	3.22
**442**	2	62	2	1	21.33333	9.333333	1	5.7	1	1.56
**451**	1	51	2	2	17.33333	10.66667	1	5.7	1	2.31
**486**	1	59	2	1	20	12	1	5.7	1	1.04
**44**	1	54	2	2	18.93333	13.46667	1	5.6	1	1.5
**78**	1	58	2	2	22.4	13.86667	1	5.6	1	1.76
**117**	1	66	2	2	22.66667	12	1	5.6	2	0.76
**60**	1	66	2	2	27.33333	14	1	5.6	1	1.59
**209**	2	70	2	1	20.26667	10.4	1	5.6	1	3.04
**47**	1	62	2	1	18.4	11.33333	1	5.6	1	0.74
**48**	1	41	2	2	19.46667	11.86667	1	5.6	1	3.16
**432**	1	37	2	1	18.66667	10.66667	1	5.6	2	3.45
**374**	1	71	2	1	22.66667	13.33333	1	5.6	1	0.83

**114**	2	39	2	1	15.46667	10.66667	1	5.6	1	0.5
**398**	1	48	2	2	22.93333	14.93333	1	5.6	1	1.08
**337**	2	59	2	2	22.26667	12	1	5.5	2	0.9
**466**	2	57	2	1	16.66667	10.66667	1	5.5	1	1.59
**341**	2	64	2	2	20	13.33333	1	5.5	1	0.79
**270**	2	65	2	2	22.66667	13.73333	2	5.5	1	1.79
**211**	2	59	2	1	21.86667	11.33333	1	5.5	2	1.39
**216**	1	57	2	2	24	12	2	5.5	2	2.14
**217**	2	54	2	1	22.4	10.66667	1	5.5	1	0.96
**406**	1	69	2	2	18.93333	9.066667	1	5.5	1	1.88
**237**	1	59	2	1	16.66667	10.66667	1	5.5	2	1.65
**243**	1	47	2	2	18.13333	11.46667	1	5.5	1	4.54
**134**	1	31	2	2	17.33333	12	1	5.5	1	1.99
**257**	1	43	2	1	20.53333	14.26667	1	5.5	2	2.92
**299**	2	78	2	2	21.33333	13.33333	1	5.5	2	1.02
**320**	1	39	2	1	21.06667	13.73333	1	5.5	1	3.02
**371**	2	72	2	2	20.53333	10.66667	1	5.5	2	1.55
**376**	1	69	2	2	21.33333	12	2	5.5	2	2.48
**378**	2	67	2	2	21.2	13.33333	1	5.5	1	1.01
**331**	1	48	2	1	14	10	1	5.5	1	1.28
**395**	1	53	2	2	19.2	12	1	5.5	1	5.14
**396**	1	51	2	2	24	16	1	5.5	2	1.58
**265**	1	52	2	1	17.33333	10.66667	1	5.4	2	1.65
**36**	1	58	2	2	24.53333	13.86667	2	5.4	2	1.5
**201**	1	57	2	1	20.13333	12.13333	1	5.4	1	4.94
**344**	1	65	2	1	22.4	13.46667	1	5.4	2	2.4
**119**	1	55	2	2	19.73333	13.33333	1	5.4	2	3.04
**170**	1	68	2	1	16	10.66667	1	5.4	2	0.63
**218**	2	50	2	2	22.53333	13.33333	2	5.4	2	1.87
**409**	1	55	2	1	18.66667	10.66667	1	5.4	2	1.03
**70**	1	46	2	1	22.66667	14.13333	1	5.4	1	1.72
**242**	1	49	2	1	26.13333	16.66667	1	5.4	1	0.68
**182**	2	76	2	1	16	10.66667	1	5.4	2	1.91
**372**	1	72	2	2	14.26667	8	1	5.4	1	1.51
**383**	1	58	1	2	22.66667	12.26667	1	5.4	1	1
**438**	1	67	1	2	22.66667	14.66667	1	5.4	1	1.82
**449**	1	53	2	2	27.73333	17.06667	1	5.4	1	1.29
**135**	1	59	2	1	19.46667	9.333333	1	5.4	1	1.3
**394**	2	59	2	1	21.33333	13.33333	1	5.4	1	0.8
**7**	1	56	2	2	22.26667	14	1	5.3	1	2.4
**58**	1	59	2	1	20.8	12	1	5.3	1	1.28
**196**	1	60	2	2	21.33333	10.66667	1	5.3	2	1.62
**469**	1	66	2	2	23.46667	11.86667	1	5.3	2	1.21
**403**	1	59	2	1	20.53333	12	1	5.3	1	3.18
**481**	1	56	2	2	21.33333	10.66667	1	5.3	2	2.42
**63**	2	74	2	1	18	11.86667	1	5.3	1	1.22
**220**	1	68	2	2	23.73333	13.86667	1	5.3	1	1.02
**357**	1	54	2	1	19.6	13.33333	1	5.3	1	1.08

**224**	1	65	2	2	24.66667	16	1	5.3	1	2.24
**149**	2	67	2	2	17.33333	10.66667	1	5.3	2	2.69
**236**	1	59	2	2	25.73333	13.06667	1	5.3	1	0.6
**288**	2	68	2	2	20	10.66667	1	5.3	1	1.85
**426**	2	55	2	2	20	10.66667	2	5.3	1	0.9
**89**	1	63	1	2	17.33333	10.66667	1	5.3	2	2.39
**305**	1	65	2	2	22.26667	11.2	1	5.3	1	3.71
**306**	1	64	2	2	18.66667	10.66667	1	5.3	1	1.02
**307**	1	64	2	2	19.06667	11.73333	1	5.3	2	0.96
**443**	1	59	2	2	17.46667	11.33333	1	5.3	1	1.52
**458**	2	76	2	2	20.66667	11.33333	1	5.3	2	1.73
**459**	1	64	2	2	21.33333	10.66667	1	5.3	2	1.36
**461**	1	58	2	1	18.53333	10	1	5.3	1	0.84
**37**	1	73	2	2	24	12	1	5.2	2	1.88
**470**	1	59	2	2	17.73333	11.2	1	5.2	1	0.61
**402**	1	73	2	2	27.73333	14.13333	1	5.2	2	2.98
**414**	2	70	2	2	20.8	10.4	2	5.2	1	1.59
**103**	2	63	2	2	21.33333	12.66667	2	5.2	1	0.66
**289**	2	66	2	1	21.33333	12	1	5.2	1	1.18
**361**	2	65	2	1	20	13.33333	1	5.2	2	0.84
**419**	2	65	2	2	24	12	1	5.2	2	1.43
**429**	1	52	2	1	17.33333	10.66667	1	5.2	1	4.01
**433**	1	75	2	2	16	10.66667	1	5.2	2	2.53
**436**	1	72	2	2	22.13333	12.53333	1	5.2	1	1.93
**448**	2	56	2	2	23.2	13.73333	1	5.2	1	5.2
**400**	2	41	2	2	23.33333	12	1	5.2	1	1.42
**221**	2	67	2	2	22.53333	13.33333	1	5.15	1	1.14
**487**	1	60	2	1	17.73333	9.466667	1	5.11	1	0.99
**138**	2	62	2	1	21.06667	10.4	1	5.1	1	0.45
**162**	1	77	2	2	20.8	10.66667	1	5.1	1	1.41
**264**	1	57	2	2	22.66667	12.8	1	5.1	2	1.94
**59**	2	57	2	2	20.66667	14	1	5.1	2	0.99
**25**	1	50	2	1	16	9.333333	1	5.1	1	1.53
**210**	1	60	2	2	22.26667	12	1	5.1	2	2.22
**226**	1	59	2	1	26.66667	14.66667	1	5.1	1	0.72
**0**	1	38	1	2	22	16	2	5.1	1	1.39
**42**	2	57	2	2	22.4	10.93333	1	5.1	1	1.22
**102**	2	64	2	1	17.06667	10.66667	1	5.1	2	1.5
**15**	1	55	2	1	22.53333	12	1	5.1	2	0.86
**301**	2	70	2	2	21.6	11.06667	1	5.1	1	1.72
**319**	1	40	2	1	18.8	13.46667	1	5.1	2	1.61
**263**	1	53	2	1	19.86667	10.66667	1	5	2	1.8
**20**	1	65	2	2	20.66667	14	1	5	2	1.34
**56**	1	60	2	1	16.26667	12	1	5	1	1
**93**	1	49	2	2	21.33333	13.73333	1	5	1	1.29
**198**	2	78	2	2	21.33333	12	1	5	1	1.8
**471**	1	64	2	1	17.6	12	1	5	1	3.7
**207**	1	75	2	2	22	10.66667	2	5	2	0.66

**206**	2	76	2	2	26.8	9.866667	1	5	1	1.31
**145**	1	76	2	1	24	13.33333	1	5	1	1.45
**49**	1	51	2	2	23.33333	12.66667	1	5	2	1.99
**84**	1	60	2	2	22.53333	12	1	5	1	1.12
**219**	1	69	2	2	23.46667	13.6	1	5	1	1.46
**355**	1	61	2	2	22.66667	12.8	1	5	2	2.13
**105**	2	58	2	1	24.8	12.66667	1	5	2	0.61
**131**	1	57	2	2	18.4	10.66667	2	5	1	1.33
**421**	1	64	2	2	24	12	1	5	2	0.83
**52**	1	66	2	1	23.46667	12.8	2	5	2	1.96
**133**	1	47	2	1	20	10.66667	1	5	1	1.57
**159**	1	60	2	1	18.66667	12	1	5	1	3.15
**187**	2	53	2	2	20	13.33333	1	5	1	5.45
**251**	2	61	2	2	19.33333	13.33333	1	5	1	1.38
**303**	1	69	2	2	22.13333	12	1	5	2	0.87
**379**	1	64	2	1	19.46667	14.4	1	5	1	2.4
**434**	1	74	2	2	19.73333	12	1	5	1	1.08
**456**	2	37	2	2	18.4	8.933333	1	5	1	1.73
**477**	1	68	2	1	20	12	1	5	2	0.65
**113**	1	50	2	1	17.86667	12.93333	1	5	1	0.7
**137**	1	61	2	2	18.8	10.66667	2	4.9	1	0.71
**203**	2	63	2	2	18.93333	10.93333	1	4.9	2	1.34
**268**	1	69	2	2	17.33333	10.66667	1	4.9	1	2.11
**65**	1	58	2	1	16	9.333333	1	4.9	2	0.73
**124**	1	43	2	1	16.66667	11.2	1	4.9	1	0.82
**14**	1	64	2	2	18.66667	12	1	4.9	2	1.22
**179**	1	60	2	1	22	13.2	1	4.9	1	2.26
**51**	1	62	2	2	22.66667	12	1	4.9	1	1.54
**129**	1	60	2	1	16	8	1	4.9	1	1.23
**152**	1	59	2	1	20.13333	12	1	4.9	1	2.48
**287**	1	69	2	2	21.2	13.2	1	4.9	2	1.68
**363**	1	58	2	2	18.66667	10.66667	1	4.9	1	2.62
**417**	1	71	2	1	22.93333	12	1	4.9	1	0.95
**53**	2	58	2	2	24	12	1	4.9	1	5.65
**110**	1	52	2	1	19.86667	12.26667	1	4.9	1	2.1
**435**	2	73	2	1	18	9.333333	1	4.9	1	3.63
**478**	2	67	2	1	19.73333	9.333333	1	4.9	1	0.87
**484**	2	53	2	2	18.66667	11.73333	1	4.9	1	1.86
**191**	1	62	2	1	20	10.66667	1	4.9	1	0.62
**325**	2	65	2	2	20.53333	13.46667	1	4.9	1	1.57
**6**	2	69	2	2	24	13.33333	1	4.8	1	0.84
**31**	1	73	2	2	21.33333	13.33333	1	4.8	1	1.03
**480**	1	75	2	2	17.33333	10.66667	1	4.8	1	1
**77**	2	62	2	1	17.33333	10.66667	1	4.8	2	1.68
**197**	1	52	2	2	22.66667	16	1	4.8	1	1.21
**139**	1	52	2	1	20.8	11.86667	1	4.8	1	2.53
**171**	1	67	2	2	21.33333	12	1	4.8	2	1.77
**172**	1	60	2	2	22.66667	13.33333	1	4.8	1	0.81

**173**	2	52	2	2	22.13333	14.4	1	4.8	1	1.25
**272**	2	57	2	1	18.66667	11.33333	1	4.8	1	1.32
**345**	2	68	2	1	24	13.33333	1	4.8	2	2.39
**273**	1	78	2	2	17.33333	10.4	1	4.8	2	1.15
**225**	1	64	2	1	28	14.66667	1	4.8	1	0.9
**280**	1	70	2	2	21.33333	12	1	4.8	2	1.95
**356**	1	61	2	2	22.66667	12	1	4.8	2	1.59
**86**	1	63	2	1	14.4	10.66667	1	4.8	2	1.73
**239**	2	53	2	2	20.8	12	1	4.8	2	1.08
**291**	2	62	2	1	18.66667	10.66667	1	4.8	1	1.58
**39**	1	64	2	1	18.66667	10.66667	1	4.8	1	1.45
**91**	1	45	2	1	21.33333	12	1	4.8	2	2.1
**155**	2	72	2	2	20	13.33333	1	4.8	1	4.4
**184**	2	65	1	2	24	13.33333	1	4.8	1	1.26
**244**	1	78	2	2	20.93333	9.6	1	4.8	2	1.83
**246**	1	70	2	1	21.73333	9.733333	1	4.8	2	1.86
**308**	1	63	2	1	17.6	10.53333	1	4.8	2	0.82
**375**	1	70	1	2	24	13.33333	1	4.8	1	1.59
**387**	1	52	2	1	19.86667	11.86667	1	4.8	1	2.28
**444**	1	59	2	1	18.53333	12.66667	1	4.8	1	1.1
**260**	1	58	2	1	18.66667	14.66667	1	4.8	1	2.27
**165**	1	48	2	2	20.66667	12	1	4.7	2	1.71
**195**	1	73	2	2	16	10.66667	1	4.7	1	1.31
**83**	1	63	2	1	17.33333	10.66667	1	4.7	1	0.82
**285**	1	57	2	1	16.93333	11.33333	1	4.7	2	1.08
**71**	1	45	2	2	17.33333	12	1	4.7	2	1.84
**106**	1	54	2	2	21.33333	12	2	4.7	1	0.92
**107**	1	42	2	2	25.33333	17.33333	1	4.7	1	1.38
**294**	1	53	2	2	16	10.66667	1	4.7	1	1.53
**427**	1	53	2	1	15.2	9.866667	1	4.7	1	0.62
**476**	1	54	2	2	18.13333	10.66667	1	4.7	1	5.04
**249**	2	65	2	1	20	10.66667	1	4.7	1	0.83
**315**	1	49	2	1	22.66667	13.33333	1	4.7	1	3.03
**318**	1	44	2	1	22.66667	14.13333	1	4.7	1	2.13
**368**	1	78	2	2	18.66667	9.333333	1	4.7	1	0.79
**370**	1	73	2	2	21.33333	13.33333	1	4.7	2	1.11
**388**	1	51	2	2	17.33333	12	1	4.7	2	1.89
**439**	1	67	2	2	20	10.66667	1	4.7	2	2
**450**	1	52	2	1	20	12	1	4.7	1	1.25
**321**	1	70	2	1	20.66667	13.33333	1	4.7	2	2.11
**322**	1	69	2	2	21.33333	12	2	4.7	1	0.91
**397**	2	49	2	2	24	13.33333	1	4.7	1	1.65
**462**	1	57	2	2	18.53333	12.8	1	4.7	1	0.87
**482**	1	62	2	1	17.33333	10.66667	1	4.7	2	2.12
**342**	1	77	2	2	18.66667	9.333333	1	4.6	1	0.88
**81**	1	42	2	1	18.66667	12	1	4.6	2	1.24
**96**	1	70	2	2	22.66667	12	1	4.6	2	1
**174**	1	70	2	2	23.6	13.46667	1	4.6	1	0.85

**276**	1	54	2	2	24.4	14.66667	1	4.6	2	1.28
**278**	1	49	2	2	20.66667	14	2	4.6	2	0.75
**222**	2	66	2	2	19.2	11.46667	1	4.6	1	2.33
**286**	1	49	2	1	21.33333	12	1	4.6	2	2.01
**352**	1	67	2	2	18.26667	9.333333	1	4.6	1	2.79
**354**	1	62	2	1	14.66667	10.66667	1	4.6	2	1.45
**231**	1	70	2	2	18.66667	10.66667	1	4.6	1	2.43
**88**	1	65	2	1	18.93333	11.46667	1	4.6	2	1.12
**158**	1	65	2	2	20	12	1	4.6	2	1.96
**252**	1	59	2	2	20	12	1	4.6	2	1.05
**310**	2	60	2	1	20.8	8.533333	1	4.6	2	1.09
**313**	1	55	2	2	20.4	13.33333	1	4.6	2	1.43
**316**	2	47	2	1	18.13333	10	1	4.6	1	0.63
**389**	1	48	2	1	21.33333	13.33333	1	4.6	2	3.77
**479**	1	50	2	1	23.46667	14.66667	1	4.6	2	1.45
**112**	1	52	2	2	22.53333	13.33333	1	4.6	2	0.7
**192**	1	58	2	2	18.66667	10.66667	1	4.6	2	1.72
**259**	1	61	2	1	16	10.66667	1	4.6	1	1.7
**391**	1	73	2	1	17.33333	10.66667	1	4.6	1	0.57
**54**	1	38	2	1	19.6	10.66667	1	4.5	1	0.92
**200**	1	65	2	1	23.46667	13.06667	1	4.5	1	1.22
**202**	1	64	2	2	22.66667	14	1	4.5	2	1.82
**142**	1	60	2	2	24	13.33333	1	4.5	2	3.45
**277**	1	53	2	1	24	13.73333	1	4.5	1	1.47
**408**	2	57	2	1	22.26667	10.66667	1	4.5	2	1.34
**126**	1	59	2	2	16	10.66667	1	4.5	1	0.7
**359**	1	42	2	1	20	10.66667	1	4.5	1	1.7
**415**	1	66	2	1	17.6	10.66667	1	4.5	1	0.8
**241**	1	49	2	2	23.73333	15.33333	1	4.5	2	1.38
**296**	2	50	2	1	15.33333	10.66667	1	4.5	2	0.91
**188**	1	51	2	2	21.33333	14.66667	1	4.5	2	2.37
**254**	1	52	2	2	22.66667	13.33333	1	4.5	2	3.26
**381**	1	59	2	2	22.66667	13.33333	1	4.5	1	1.56
**440**	2	66	2	1	21.06667	13.2	1	4.5	2	0.82
**336**	1	32	2	1	18.8	10.66667	1	4.5	1	0.77
**465**	1	32	2	1	18.13333	10.66667	1	4.5	2	0.59
**29**	1	37	2	2	18.93333	13.06667	1	4.4	2	0.96
**95**	1	45	2	2	20.26667	13.86667	1	4.4	1	0.74
**169**	2	76	1	2	17.33333	10.66667	1	4.4	2	2.76
**80**	1	55	2	2	23.2	13.73333	1	4.4	2	1.34
**97**	1	50	2	2	20	12	1	4.4	1	0.7
**41**	1	58	2	2	21.06667	11.73333	1	4.4	1	1.08
**178**	1	55	2	1	17.73333	10.4	1	4.4	1	1.37
**215**	1	62	2	1	17.33333	10.66667	1	4.4	1	1.2
**348**	1	67	2	2	21.86667	13.2	1	4.4	1	1.09
**412**	1	43	2	1	16	9.333333	1	4.4	2	3.03
**180**	1	35	2	1	18.66667	10.66667	1	4.4	2	2.47
**108**	1	26	2	1	17.33333	10.66667	1	4.4	1	0.64

**233**	1	67	2	1	18.66667	10.26667	1	4.4	2	2.64
**290**	1	66	2	2	21.73333	13.2	1	4.4	2	4
**297**	1	47	2	2	20.26667	10.53333	1	4.4	2	0.72
**437**	2	70	2	1	20	13.33333	1	4.4	1	0.88
**454**	1	41	2	2	20	13.33333	1	4.4	2	1.27
**327**	1	59	2	1	14.66667	10.66667	1	4.4	1	1.51
**390**	1	74	2	2	21.33333	12.66667	1	4.4	2	1.94
**340**	1	69	2	2	22	12	1	4.3	1	1.35
**267**	2	79	2	2	20.8	10.66667	1	4.3	1	1.92
**473**	1	59	2	1	22.4	14.66667	1	4.3	1	1.58
**279**	1	46	2	2	22	14.8	1	4.3	2	2.21
**350**	1	47	2	2	18.66667	10.66667	1	4.3	1	1.92
**410**	1	49	2	2	18.93333	13.73333	1	4.3	1	1.57
**413**	1	74	2	1	23.2	12.53333	1	4.3	2	0.91
**73**	1	43	2	2	21.6	13.86667	1	4.3	2	1.77
**90**	1	55	2	2	16.53333	10.66667	1	4.3	1	0.8
**256**	2	46	2	2	26.66667	16	1	4.3	1	1.92
**302**	1	70	2	1	17.2	10.53333	1	4.3	1	0.83
**367**	1	80	2	1	21.33333	10.66667	1	4.3	2	0.57
**262**	1	43	2	1	18.66667	10.66667	1	4.3	2	1.97
**485**	1	79	2	2	18.66667	10.66667	1	4.3	2	1.09
**24**	2	36	2	2	21.73333	15.2	1	4.23	1	0.74
**34**	1	53	2	1	19.2	12.53333	1	4.2	1	0.84
**116**	1	51	2	2	23.33333	14.26667	1	4.2	1	1.07
**128**	2	66	2	2	18.66667	10.66667	2	4.2	1	2.63
**364**	1	55	2	1	19.73333	11.33333	1	4.2	2	5.6
**250**	1	62	2	2	21.33333	12	1	4.2	1	1.05
**160**	1	65	2	1	18.66667	12	1	4.2	1	0.97
**328**	1	58	2	1	22.4	13.73333	1	4.2	1	1.54
**335**	1	39	2	1	18.66667	10.66667	1	4.2	1	2.07
**472**	1	46	2	2	25.33333	13.33333	1	4.1	2	4.2
**176**	1	68	2	2	16.4	10.26667	1	4.1	1	1.02
**68**	1	53	2	1	19.73333	12	1	4.1	2	0.78
**366**	1	48	2	2	21.33333	13.6	1	4.1	1	1.2
**16**	1	52	2	2	16	9.466667	1	4.1	1	1.38
**447**	1	57	2	2	18.66667	12.66667	1	4.1	1	2.24
**2**	1	55	2	1	20	13.06667	1	4.1	1	1.76
**332**	1	48	2	1	16	9.333333	1	4.1	1	0.68
**43**	1	57	2	2	24	13.33333	1	4	2	1.72
**23**	1	65	2	2	22.13333	11.06667	1	4	2	1.47
**82**	1	73	2	1	21.33333	12	1	4	1	0.89
**312**	1	58	2	2	20.4	13.6	1	4	1	1.34
**411**	1	48	2	2	24	13.33333	1	3.9	2	2.37
**101**	2	70	2	1	24	12	1	3.9	1	1.69
**189**	1	43	2	2	18	9.333333	1	3.9	1	1.26
**446**	1	57	2	1	17.33333	12.66667	1	3.9	2	5.41
**55**	1	43	2	2	21.73333	12.93333	1	3.8	1	1.86
**50**	1	41	2	2	18	12	1	3.8	2	1.66

**295**	2	51	2	2	21.33333	13.33333	1	3.8	1	3.74
**258**	1	72	2	2	18.4	10.66667	2	3.8	1	1.03
**148**	1	68	2	2	22.26667	12.4	1	3.7	1	0.92
**19**	1	39	2	2	20.66667	12.4	1	3.7	2	3
**317**	2	46	2	2	23.86667	11.73333	1	3.7	1	2.12
**416**	1	63	2	2	20.53333	12	2	3.6	2	1.04
**431**	1	50	2	1	18.66667	10.66667	1	3.6	2	0.92
**38**	1	38	2	1	19.2	12	1	3.46	1	1.17
**185**	2	56	2	2	18.13333	12.26667	1	0.9	1	4.19

C	DL	TOA	OC	Onset	DNT	OTT	NIH	mRs
3.37	1.7	3	1	2.083333	1.2	3.5	4	1
3.29	2.3	3	1	7	2	9	5	0
4.17	2.7	3	1	10	1	11	9	1
3.55	0.76	3	2	1.333333	0.8	2.716666667	10	0
5.25	3.9	3	1	3.5	2.666667	6.166666667	12	1
5.12	3.8	3	1	0.833333	1.116667	1.95	9	0
4.29	2.3	3	1	5.75	0.933333	2.433333333	5	0
6.92	4.5	3	1	6.5	2.75	9.25	4	0
5.36	3.3	3	2	3.3	1	4.3	7	1
4.15	2.7	3	1	6.166667	2.05	3	4	0
6.25	4.7	2	1	1.166667	1.583333	2.75	16	1
4.39	2.6	1	1	1.5	1.733333	3.233333333	11	1
4.95	1.62	3	1	0.583333	1.166667	1.25	20	0
3.67	1.06	3	2	6	1.133333	7.133333333	7	0
4.99	3.7	3	1	4.983333	1.416667	1.75	5	1
5.36	3.4	3	2	2	0.85	5.333333333	3	0
4.36	1.18	3	1	1.083333	1.916667	3	5	0
5.07	0.8	3	2	3.916667	1.166667	5.083333333	7	0
6.99	5.1	3	1	1.533333	1.083333	3.416666667	18	0
5.09	3.4	3	1	1.833333	1.5	3.333333333	4	0
4.68	0.74	3	1	3.55	0.7	4.25	3	1
4.22	2.6	3	2	0.333333	1	4.083333333	3	1
4.86	2.7	3	2	4.75	0.916667	4.583333333	6	0
3.38	2.3	3	4	1	2.7	3.45	8	1
4.73	3.4	3	4	2	0.916667	2.916666667	24	1
3.52	0.99	3	1	4	1.5	5.5	7	0
4.21	2.6	3	2	4.583333	1.116667	5.7	5	0
4.58	3.6	3	1	2	2.833333	6.5	6	0
4.66	3.1	3	1	0.833333	1	1.833333333	5	0
3.18	1.8	3	1	5	1	6	13	1
4.51	2.8	3	2	0.916667	1.033333	2.083333333	19	0
4.02	2.7	2	1	0.5	0.95	3.033333333	5	0
6.84	1.9	3	3	1.666667	0.733333	3.266666667	5	0
4.58	2.9	3	2	0.95	1.083333	1.75	4	0
3.66	2.3	1	1	2.166667	1.083333	1.583333333	4	1
4.34	1.6	3	1	3	1.5	4.5	8	0
4.27	0.63	3	1	1	1.5	2.5	9	0
3.92	1.5	3	4	3	1.6	2.433333333	7	1
4.79	2.9	3	1	8	0.75	8.75	20	1
5.6	1.74	3	1	4.5	0.833333	5.333333333	5	0
6.4	4.9	3	2	4.833333	1.25	3.75	4	0
5.52	1.12	3	2	1.333333	3.666667	5	9	0
3.78	2.4	3	1	2.666667	1.5	4.166666667	7	1
5.96	4.3	3	1	5	2.833333	7.833333333	14	1
3.93	0.81	3	1	0.333333	0.983333	1.316666667	6	0
5.35	3.5	3	2	4.483333	0.966667	2.166666667	6	0
4.63	3.5	3	1	1	3.75	4.75	19	1

4.4	3.1	3	1	2.133333	0.716667	9.466666667	10	0
4.12	2.7	3	1	3	2.5	5.5	8	1
3.59	2.4	3	2	3.333333	0.833333	1.5	6	0
5.57	0.9	2	2	1.5	1.5	3	7	0
5.29	3.6	3	1	6.666667	0.75	7.416666667	5	1
5.57	1.29	3	1	2	1.083333	3.083333333	10	1
4.04	2.5	3	1	1.5	0.666667	2.166666667	7	0
4.28	2.7	3	2	0.5	1.75	2.25	8	0
7.39	1.53	3	1	3	1.5	4.5	7	0
3.92	2.6	3	1	1.7	0.7	1.616666667	6	1
4.77	3.5	2	2	3.883333	1.283333	5.166666667	7	0
5.23	1.2	2	1	1.166667	1.333333	2.5	5	0
5.88	4.3	3	1	2.5	0.866667	3.366666667	20	1
6.56	4.3	3	1	1.5	2.5	4	5	1
4.28	1.22	3	1	2.25	1.666667	3.916666667	11	0
4.3	0.96	3	1	2	1.5	3.5	4	0
4.66	2.9	3	2	0.666667	3.083333	3.75	9	0
2.75	1.2	3	1	5.666667	1.25	2.5	6	0
3.91	1.52	3	4	0.583333	1.616667	2.2	21	0
4.34	2.8	3	1	2.5	1	3.5	9	0
5.43	0.91	3	1	3	1	4	6	0
5.62	3.4	3	1	2.25	6.75	9	18	1
4.8	3.2	3	1	2	2.166667	4.166666667	21	1
3.59	2.4	2	2	2.25	1.15	2.5	11	0
3.37	2.2	3	1	0.5	0.833333	1.333333333	8	1
5.19	2	3	1	3	2	5	9	1
5.97	4.1	3	3	0.583333	1	6.033333333	9	0
1.92	0.93	3	1	1	2	3.5	4	0
3.01	1.8	3	4	2.666667	1	1.833333333	12	0
5.32	1.22	3	1	3	0.75	3.75	5	0
4.51	3.2	4	1	3.666667	0.866667	1.866666667	12	1
3.95	3.1	3	2	1.683333	1.016667	2.7	9	0
3.81	2.7	2	2	1.666667	1.5	3.166666667	7	0
4.11	3	3	2	1.583333	1	5.833333333	6	0
3.95	1.05	3	4	1.5	2.333333	3.833333333	18	1
4.19	1.42	3	1	1	1.833333	2.833333333	8	0
5.07	3.9	3	1	1	0.816667	4.316666667	4	1
4.5	3.5	3	1	1	1.416667	2.416666667	10	0
1.34	3.3	3	1	2	2	5	18	0
5.9	1.53	3	1	2.25	1.666667	3.916666667	7	0
4.85	3.2	2	1	0.616667	1.616667	2.233333333	4	0
4.06	2.9	3	1	5.166667	3.416667	8.583333333	11	1
4.8	3.3	3	1	2.583333	0.75	3.333333333	7	0
5.73	1.19	3	1	3.5	1.75	5.25	7	1
4.94	3	3	2	1.75	2.166667	6.333333333	13	0
4.46	3	2	3	1.166667	1.5	2.666666667	6	0
2.22	1.4	2	2	4.166667	1.416667	5.583333333	8	0
1.32	3.4	3	1	5	1	6.5	8	0

4.18	1.08	3	1	2.25	1.833333	4.083333333	9	0
4.49	0.93	3	1	8.666667	1.416667	10.08333333	7	0
4.97	3.3	3	2	0.75	2	2.75	8	0
5.43	3.6	3	4	5	3.583333	8.583333333	23	1
5.25	4.2	3	1	0.75	1.066667	2.066666667	13	0
5.84	3.5	3	1	4	1	5.75	8	0
3.94	2.6	3	1	10.66667	3.083333	13.75	6	0
4.88	2.7	3	2	1.416667	2	3.416666667	14	0
5.84	4.1	3	1	2.5	1.333333	3.833333333	10	1
3.39	1.9	3	2	8.75	3.25	4.25	10	0
4.77	3.5	3	1	2.25	1.25	3.5	13	0
5.07	0.84	3	1	1.916667	1.8	3.716666667	6	0
4.16	2.6	3	4	1.75	1.516667	3.05	10	1
6.58	4.4	3	1	0.333333	1.4	1.733333333	20	1
5.3	1.66	3	1	1.733333	1.266667	3	13	0
5.62	4	4	1	3.166667	1.3	3.3	7	0
5.08	3.1	3	1	6	3	9	13	1
4.71	1.13	2	1	3.25	1.333333	4.583333333	10	0
5	1.15	3	1	0.583333	2.333333	2.916666667	7	0
4.16	1.41	3	1	2.166667	2.166667	4.333333333	4	0
0.52	3	3	1	1	1.05	4.55	11	0
5.95	0.96	3	1	0.5	2.166667	2.666666667	4	0
3.89	2.2	1	2	2.583333	0.833333	5.083333333	4	0
4.44	2.9	3	1	2.5	1.5	3.416666667	3	0
4.89	3.6	3	2	0.583333	1.25	2.083333333	7	0
5.08	3.4	3	1	1.25	2.083333	3.333333333	4	0
3.27	1.9	3	1	2.5	1.916667	4.416666667	4	0
4.21	1	3	1	1	1.833333	2.833333333	5	0
4.05	3.2	3	1	1.833333	1.5	3.333333333	1	0
3.67	1.7	3	1	2.166667	2.75	4.916666667	7	0
3.59	1.81	3	1	2.5	1.066667	3.566666667	4	0
4.92	3.4	3	1	1.916667	1.083333	1.916666667	8	1
8.51	7.2	3	2	1.416667	1.25	2.666666667	6	0
2.49	1.5	1	2	3.333333	1.25	4.583333333	7	0
2.56	2.2	3	1	3	0.966667	1.966666667	5	0
4.13	1.6	1	1	3.216667	1.283333	4.5	10	0
5.05	1.2	3	1	1.5	2.583333	4.083333333	6	0
4.79	1.21	3	2	0.6	0.95	2.116666667	6	1
6.31	5.4	2	1	0.95	0.933333	5.583333333	5	1
3.69	2.7	3	1	4	2.916667	6.916666667	10	1
3.52	1.9	3	1	1.5	1.416667	2.916666667	12	0
3.53	2.1	3	1	3	1.5	4.5	13	0
4.16	2.7	3	1	5.5	3.25	8.75	13	0
5.03	2.6	3	1	0.75	0.766667	1.766666667	17	0
2.47	2.9	3	1	1	1.25	5.25	11	0
2.38	1.9	2	1	0.5	1.333333	1.833333333	7	0
4.4	1.47	3	1	3	1.5	4.5	5	0
4.65	2.7	3	2	1.533333	1.166667	2.7	4	0

4.31	3.1	3	4	3	1.083333	3.15	16	1
4.74	1.69	3	1	1.083333	1.966667	3.05	22	1
5.41	4	4	1	2.5	0.95	1.366666667	7	0
2.67	1.6	3	2	0.5	0.75	2.75	6	0
4.74	3.4	3	1	1.666667	1.333333	3	6	0
5.26	3.5	3	1	0.666667	0.916667	1.833333333	6	0
4.23	2.9	3	1	3.416667	2.666667	6.083333333	7	1
4.96	3.6	3	1	0.75	1.583333	2.333333333	12	0
0.85	14.1	3	1	4	0.766667	2.766666667	5	0
3.29	2	3	1	2	2.5	4.5	5	0
3.89	3.3	3	1	2.533333	0.766667	3.3	4	0
5.96	3.9	3	2	1.5	1.666667	3.166666667	8	0
5.09	3.4	3	1	2.766667	1.183333	2.6	30	0
3.23	1.9	3	1	4	4.583333	8.583333333	4	0
5.86	1.06	3	1	1.166667	1.416667	2.583333333	5	0
3.77	2.2	3	1	8	0.5	8.5	6	0
4.88	3.3	3	2	2.583333	2.416667	5	5	0
6.34	1.2	3	1	0.666667	1.25	1.916666667	17	1
4.66	3.2	4	1	3.5	1.5	2.166666667	7	1
6.34	4.5	3	1	8.666667	0.766667	9.433333333	7	1
4.28	2.4	3	2	5.416667	0.933333	2.633333333	6	0
4.8	1	3	1	3.166667	1.833333	5	5	0
4.93	3.9	3	1	8	0.5	8.5	11	0
5.93	0.95	3	1	3	2.916667	5.916666667	5	0
4.28	2.7	3	1	3	2.6	5.6	13	0
4.34	3	3	1	8.366667	1.633333	10	10	0
4.51	3.3	3	1	1	1	4.5	9	0
4.55	1.04	3	1	2	1.333333	3.333333333	6	0
5.77	2.11	3	1	0.666667	1.3	1.966666667	6	0
3.66	2.5	2	3	3.583333	4.25	4.75	4	0
3.89	2.8	3	1	2.3	1.283333	3.583333333	17	1
4.02	2.2	3	4	1.25	2	3.25	15	1
5.31	4	3	2	3.783333	1.3	5.083333333	6	0
4.77	1.34	3	1	1.166667	1.5	2.666666667	11	0
3.85	2.9	3	1	0.833333	1.166667	2	9	0
6.46	3.5	3	1	3	1	4	6	0
4.77	2.6	2	1	5.5	2.2	7.7	4	0
5.5	4.1	3	1	2.416667	1.333333	7.5	4	0
4.71	4	3	1	3.25	2.05	5.3	1	0
6.69	4.6	3	1	2	1.5	3.5	14	1
3.76	1.02	3	1	6	1	7	12	1
4.9	3.2	3	2	1	1.833333	2.833333333	10	1
5.61	3.6	3	1	0.666667	5.333333	6	13	1
3.04	3.04	3	1	1.516667	1.483333	3	7	0
5.18	2.9	3	1	2.833333	6.166667	9	14	0
4.47	3.2	3	1	1.25	0.916667	2.166666667	14	0
3.84	0.77	3	1	0.583333	5.516667	6.1	4	0
3.74	2.6	3	2	0.916667	1.25	3.666666667	5	0

3.61	2	3	2	0.75	1.333333	2.083333333	11	0
3.07	1.2	3	1	2.333333	1.5	3.833333333	5	0
6.25	4.8	3	1	1.416667	1.166667	2.583333333	5	1
4.53	2.9	3	1	0.5	1.166667	3.25	3	1
5.43	3.2	3	1	7.5	2.133333	9.633333333	5	1
5.02	3.5	3	3	1.666667	1.583333	3.25	6	0
6.1	4.4	3	2	4.833333	1	5.833333333	7	1
7.26	5.5	3	3	0.983333	0.95	1.566666667	7	0
4.43	3.2	3	1	0.75	1	7	7	0
4.84	3.6	3	2	2.416667	1.283333	2.2	4	0
4.41	3	3	1	0.833333	1.583333	3.916666667	7	0
5.25	0.85	3	1	2	1.166667	3.166666667	7	0
3.87	1.01	2	2	1	1.4	2.4	10	0
3.73	2.3	3	1	3.833333	1.083333	3.666666667	7	0
4.24	3	3	1	1	1.666667	2.666666667	6	0
1.97	1.9	3	1	5.5	1.5	2.5	6	0
5.54	4.2	2	2	2	1.383333	3.383333333	5	0
5.77	4.2	3	1	1.033333	2.8	3.833333333	5	0
3.59	1.81	3	1	0.5	2.083333	2.583333333	5	0
4.45	2.7	3	1	1.166667	1.333333	2.5	6	0
4.14	0.75	3	2	4	2	6	5	0
6.33	4.6	3	2	0.75	1.9	4.666666667	5	0
5.87	4.3	3	1	0.5	1.466667	6.05	6	0
6.4	1.2	3	1	2	1.666667	3.666666667	16	1
5.78	3.5	3	1	0.5	1.216667	1.716666667	7	1
5.09	3.6	3	1	0.916667	0.55	1.666666667	5	1
4.58	2.7	3	1	0.833333	2.416667	3.25	10	1
3.52	2.1	3	2	1	0.85	2.566666667	8	1
4.45	1.04	3	1	2	1.25	3.25	7	0
3.36	2.2	3	1	0.083333	0.083333	0.083333333	4	0
5.54	1.35	3	1	2	1.75	3.75	13	0
3.27	1.7	3	2	4	2	6	7	0
4.53	3.1	3	2	4.5	1.5	6	8	0
3.51	0.92	3	2	2	1.083333	3.083333333	5	0
2.69	1.1	3	1	2	2.833333	4.5	5	0
5.35	4.8	3	1	1.583333	1.283333	2.866666667	4	0
4.57	2.9	3	1	3	0.8	38.08333333	4	0
4.57	1.15	3	1	0.666667	1.333333	2	10	0
5.4	3.3	3	2	1.416667	1	2.666666667	5	0
5.48	0.84	3	1	2	2	4	21	1
4.96	3.1	3	1	3	1.5	4.5	13	1
3.66	2.4	3	4	2.166667	0.65	2.4	7	1
4.96	3.2	3	1	1.25	1.5	2.666666667	3	1
0.76	2.1	3	1	1.5	1.5	3	4	1
5.28	4	3	1	1.2	1.666667	3.166666667	2	1
5.21	3.7	3	1	6	2	8	13	0
4.94	3.4	3	1	1.5	4.166667	5.666666667	7	0
3.28	1.8	3	2	0.85	1.333333	3	5	0

7.49	4.5	3	1	1	3.166667	4.166666667	7	1
6.1	4.4	3	1	1	1.083333	5.083333333	9	0
4.78	1.9	3	1	3	2.333333	5.333333333	7	0
3.83	2.5	3	1	4	2.6	6.6	6	0
4.24	1.08	3	1	4	1	5	4	0
5.27	3.8	3	1	2.333333	0.916667	2.583333333	12	0
0.73	1.91	3	1	1.5	1.333333	1.833333333	6	0
4.09	2.8	3	2	1.5	0.916667	1.666666667	6	0
3.46	2.4	3	1	4	1.333333	5.333333333	6	0
4.81	3.1	3	2	1.833333	3.416667	5.25	4	0
4.62	2.9	1	1	5.033333	1	1.566666667	4	0
2.47	1.5	3	1	3	0.683333	3.683333333	4	0
4.31	3.3	3	1	2.4	2.083333	4.5	4	0
5.11	3.9	3	1	9.5	1.75	11.25	16	1
3.65	2.9	3	2	1.35	0.866667	2.616666667	3	1
4.77	3.1	3	1	1	0.933333	1.933333333	4	1
4.24	2.9	3	1	0.833333	1.466667	2.466666667	4	0
3.8	2	3	1	0.75	2.916667	3.666666667	11	0
4.79	1.41	3	1	3.5	1	4.5	6	0
4.47	2.8	3	1	4.583333	1.283333	5.866666667	5	0
4.7	3.1	3	1	0.4	0.666667	2.666666667	4	0
1.47	0.74	3	1	3	2	3.5	4	0
7.17	5.2	3	1	5	1.5	6.5	4	0
5.46	1.03	3	3	4	1.15	5.15	4	0
1.58	3.5	3	1	1	0.5	5.5	4	0
4.48	3.1	3	2	3.666667	1.166667	4.416666667	5	0
4.12	1.46	3	1	3.8	1.5	5.3	7	0
2.68	1	3	1	1.833333	0.833333	0.266666667	0	0
3.93	3.1	1	1	4.65	2	2.833333333	9	1
5.13	3.5	3	1	3	3.083333	6.083333333	8	1
5.25	3.4	3	1	1.133333	1.25	1.75	6	1
7.13	5.4	3	1	2.083333	1.75	2.5	13	1
3.71	1.8	3	1	1.3	5.116667	6.416666667	18	1
4.44	2.7	3	1	1.5	1.166667	2.666666667	7	1
3.48	2.4	3	1	2.166667	1.033333	3.2	7	0
4.2	2.5	3	1	0.666667	1.95	4.2	11	0
4.76	3	3	1	1.5	0.933333	1.766666667	15	0
4.61	3.2	1	1	1.166667	0.833333	4.5	11	0
3.53	2	3	1	2.916667	1	2	20	0
5.96	1.73	3	1	6.833333	0.966667	7.8	6	0
3.64	2.5	3	1	2	3	5	6	0
4.64	3.1	3	1	4.583333	2.533333	4.283333333	6	0
4.13	2.8	3	2	0.75	2.583333	4.916666667	18	1
3.49	2.3	3	1	2.166667	2.466667	4.633333333	13	0
4.89	3.3	3	1	0.666667	1.333333	2	11	1
4.63	3.2	3	1	0.833333	1	4.5	7	1
0.82	2.6	3	1	2	1.5	5.166666667	3	1
3.9	1.46	3	2	2	1.416667	3.416666667	7	0

3.14	1.7	3	1	0.566667	1.4	2.066666667	7	1
3.85	2.2	3	1	4.666667	2.166667	6.833333333	9	0
4.53	3.2	3	1	1.166667	1.083333	1.583333333	14	0
3.91	3.1	2	1	2.75	1.366667	4.116666667	12	0
2.76	1.5	3	1	4	1	5	7	0
4.77	3.2	2	3	1.166667	1.666667	2.166666667	5	1
3.99	2.6	3	1	0.5	1.3	3.3	11	0
4.56	1.6	3	2	3.866667	1.466667	5.333333333	10	0
3.75	2.4	3	1	6.333333	3.666667	10	4	0
5.09	3.5	3	1	1	1.3	6.083333333	14	0
3.49	2.6	3	1	0.833333	2.283333	4.033333333	10	0
3.78	2.4	3	1	0.5	1.033333	1.533333333	9	0
1.28	3.7	3	1	1.5	1.15	2.15	8	0
4.01	2.3	3	1	2.75	0.833333	3.583333333	7	0
3.8	1.26	3	1	2	1.3	3.3	6	0
7.53	5.3	3	1	1.333333	3.166667	4.5	5	0
3.71	2	3	1	3.5	1.033333	4.533333333	4	0
4.25	2.9	1	1	6.5	0.916667	7.416666667	4	0
5.45	3.7	3	1	1	1.366667	2.366666667	3	0
5.34	4	2	2	1.083333	1.583333	1.833333333	11	0
4.42	1.13	3	1	1	1.25	2.25	9	1
3.05	1.9	3	1	2.416667	0.966667	1.883333333	7	0
4.59	3.5	3	1	2.333333	1.5	3.833333333	6	0
5.46	4.2	3	1	3.833333	1	4.833333333	13	0
4.52	2.9	3	1	1.5	2.166667	3.666666667	10	1
7.29	5.3	1	1	2.033333	1.8	3.833333333	20	0
5.02	3.2	3	1	0.75	2.5	3.25	8	0
4.18	1.3	3	1	2.75	1.416667	4.166666667	14	0
4.37	2.6	3	1	0.5	3.333333	3.833333333	10	0
8.05	5.6	3	1	3.083333	4	7.083333333	9	0
4.28	0.94	3	1	4	1.633333	5.633333333	6	0
3.6	2	3	2	4.133333	1.5	4.833333333	5	0
3.81	2.6	3	1	1.416667	1.416667	2.833333333	4	0
1.37	1.19	3	1	1.5	2	3	14	0
4.04	2.2	2	1	8	1	9	11	0
0.63	2.1	3	1	1	0.916667	2.416666667	4	0
3.42	2.5	2	2	0.8	1.083333	3.5	3	0
3.95	2.4	3	3	2.533333	1.183333	6.166666667	1	0
2.91	1.5	2	2	4.166667	1.7	3.2	8	0
5.39	3.9	3	1	3.5	1.033333	2.283333333	6	0
4.51	3.2	3	1	3	1	3.25	22	1
3.42	2	3	2	1.75	2.666667	4.416666667	17	1
4.39	2.6	1	4	1	2.8	3.4	2	1
5.35	3.7	3	1	1.5	1.166667	2.666666667	12	1
4.08	2.8	3	1	3.25	0.833333	6.5	7	1
4.16	2.9	2	3	2.2	1.383333	3.583333333	9	1
5.79	1.31	3	1	1.05	1.416667	2.916666667	8	1
4.81	2.6	2	1	1.333333	0.966667	2.05	8	1

4.71	1.11	3	1	4	1	5	8	1
4.11	2.6	3	2	2.333333	1.25	2.083333333	6	1
4.77	3.1	3	1	3.666667	1.75	5.416666667	5	1
3.55	2.3	3	1	1	1.066667	2.566666667	6	0
4.04	1.7	3	2	4.25	1.5	5.75	7	0
4.41	3.2	3	1	6	1.066667	7.066666667	6	0
2.96	1.8	3	1	0.833333	0.666667	1.5	5	0
3.38	2.4	3	1	0.85	1.15	2	12	0
4.28	3	3	1	3.416667	1.333333	4.75	7	0
4.46	3.2	3	1	0.25	0.85	1.833333333	6	0
2.7	0.91	3	2	1.416667	1.25	2.666666667	16	0
3.91	2.5	3	1	1	1.166667	3.333333333	12	0
0.68	1.91	3	1	1	1.5	3.5	9	0
4.07	0.94	3	1	2.5	1.416667	3.916666667	8	0
5.71	1	3	2	3	1.466667	4.466666667	7	0
4.94	3.5	3	2	0.666667	1.066667	1.733333333	7	0
4.43	3.2	3	1	1.5	3.283333	0.05	6	0
3.79	0.79	3	3	3	1.333333	4.333333333	5	0
5.22	1.19	3	1	6.6	1.1	7.7	5	0
4.47	3	3	1	1.35	4.116667	6.616666667	4	0
4.67	2.9	3	1	2.5	1.333333	3.833333333	7	0
3.89	2.5	3	1	1.5	0.933333	1.6	8	1
2.36	1.2	3	1	0.75	1.25	1.666666667	7	1
4.32	2.8	3	1	6	1	2.333333333	12	0
4.58	3	2	2	1.116667	0.666667	4.25	6	0
5.47	3.7	3	1	1.5	2.416667	3.916666667	13	0
3.73	1.17	3	2	3.166667	1.25	4.416666667	11	0
4.68	3.3	3	1	0.783333	3.2	4.866666667	11	0
3.3	2.2	3	1	8	5.5	13.5	6	0
2.65	1.4	3	1	2.5	0.866667	5	4	0
5.27	0.69	3	1	0.333333	2.583333	2.916666667	3	0
3.89	1.12	3	1	2	2	4	7	0
1.09	2.1	3	1	1	1.5	4.5	6	0
4.34	3.2	3	1	0.916667	1.316667	2.233333333	6	0
4.23	3.3	1	1	1.266667	0.8	2.066666667	5	0
4.55	3.5	3	2	1.666667	1.883333	4.05	5	0
3.44	2.2	3	2	3.166667	1.416667	4.583333333	5	0
3.77	2.3	3	2	2	0.75	1.75	4	0
4.12	2.9	3	3	4.25	1.833333	6.083333333	4	0
4.19	2.8	3	2	0.6	1.366667	1.966666667	6	0
2.55	1.2	3	3	0.616667	0.833333	1.833333333	6	0
4.26	1.04	3	1	2	1.5	3.5	5	0
3.65	2.8	3	1	1.55	0.7	2.25	4	0
3.27	3.2	3	2	2.583333	1.7	4.283333333	2	0
5.41	4	3	1	1.25	1.416667	2.166666667	5	1
4.44	3.3	3	1	3	1.25	4.25	12	0
2.93	1.19	3	1	4.5	1.7	6.2	11	0
3.95	2.9	3	1	1.816667	1.016667	2.833333333	8	0

5.56	4.2	3	1	1.333333	1.116667	1.366666667	6	0
4.63	2.7	2	3	2.05	0.716667	6.716666667	6	0
6.72	1.67	3	1	8	1.166667	9.166666667	7	0
4.47	3	3	2	1.433333	1	4.166666667	6	0
3.39	2.9	3	1	1.2	1.333333	2.533333333	5	0
4.9	3.5	3	1	2.633333	1.666667	3.333333333	5	0
5.26	3.8	2	2	8.5	1.5	10	7	0
3.33	2.3	3	1	1.333333	1.2	2.533333333	12	0
3.31	2.1	3	2	5.166667	1.65	6.816666667	9	0
5.06	3.6	3	1	1.666667	1.416667	2.166666667	7	0
4.75	3.2	3	1	0.75	2.75	3.5	6	0
3.16	1.9	3	2	5.333333	1.55	2.5	6	0
3.63	1.44	3	1	1	2	3	6	0
5.78	3.7	2	2	3.5	1.666667	5.166666667	5	0
5.82	4.2	3	2	2	1.916667	6.166666667	3	0
5.38	4.1	3	2	0.416667	1.2	2.333333333	11	0
3.02	1.4	3	1	4	2.1	6.1	8	0
3.49	2	3	1	0.5	8	8.5	7	0
2.71	1.4	3	1	1.9	1.6	3.5	5	0
3.84	2.4	2	2	0.666667	2.333333	3	13	1
4.53	2.6	2	1	1.25	1.583333	2.833333333	7	0
5.27	3.9	3	2	1.916667	0.933333	1.933333333	7	1
4.53	3	3	2	1.5	1.333333	2.833333333	9	0
6.27	4.3	2	3	3.666667	1.833333	5.5	6	0
6.1	4.4	3	2	0.5	0.833333	1.333333333	4	0
2.69	1.7	1	1	0.716667	1.283333	2.666666667	10	0
4.12	2.9	3	1	1.666667	1.25	2.916666667	5	0
4.81	3.5	1	1	1.666667	3.383333	4.633333333	4	0
5.94	4.2	3	1	4.783333	1	3.916666667	7	0
3.85	2.3	3	1	6.166667	1.833333	8	6	0
4.89	0.93	3	1	1.166667	1.483333	2.65	8	0
5.1	0.88	3	1	0.833333	2.5	3.333333333	7	0
3.24	1.8	3	2	4.166667	1.15	2.316666667	5	0
4.37	2.8	3	1	0.916667	2.25	3.166666667	4	1
4.23	2.3	3	1	5	1.666667	6.666666667	6	0
2.59	1.29	3	1	2	2	4	4	0
4.7	3.3	3	1	1.5	1.666667	3.166666667	17	1
3.49	2.3	3	1	1.333333	1.15	2.483333333	11	0
4.54	3	3	1	2.833333	1.866667	4.7	8	0
4.09	0.95	3	1	7.5	1.266667	8.766666667	12	1
4.29	1.32	3	1	5	1	6	11	1
3.62	2.5	3	2	0.833333	1.166667	1.75	15	0
5.46	4.3	3	1	1.133333	0.866667	2	8	0
4.49	1.02	3	1	1	1	2	7	0
6.15	4.9	3	1	1.25	1.2	2.45	5	0
4.9	2.9	3	2	1.5	2	3.5	4	0
5.32	3.8	3	2	1.5	1.183333	1.583333333	8	0
0.64	2.5	3	1	1.5	1	2.5	11	0

4.02	2.2	2	3	1	0.85	1.35	7	0
4	1.9	3	1	0.666667	1	3.416666667	6	0
5.1	3.5	3	1	2	1.083333	2.666666667	6	0
3.97	2.8	2	2	2.166667	1.25	3.416666667	4	0
3.65	2.5	3	2	2.75	1.683333	4.433333333	4	0
3.56	2.2	3	1	1.666667	1.2	3.833333333	6	0
5.36	3.9	3	1	3.5	0.866667	1.716666667	5	0
3.98	2.6	3	1	1.5	1.083333	2.416666667	5	1
3.93	2.9	2	2	1	1.333333	2.333333333	6	0
3.99	2.8	2	1	0.25	1.366667	3.5	3	0
2.81	1.4	3	2	0.5	1.733333	5.4	6	0
4.1	2.8	3	1	3.083333	0.783333	6.533333333	5	0
5.21	3.9	3	2	2	1.416667	3.416666667	4	0
3.36	2.2	3	2	1.5	2.566667	4.066666667	4	0
6.6	4.9	3	2	0.666667	0.816667	1.233333333	13	0
4.34	2.8	3	1	0.5	0.65	2	12	0
4.95	3.5	3	1	1.416667	2.083333	3.5	7	0
4.79	3	3	1	1.25	0.666667	1.416666667	6	0
3.44	2.4	3	1	7	1.5	8.5	5	0
4.2	2.8	3	1	2	2.166667	3.666666667	7	0
4.21	2.7	2	3	2.583333	0.75	2.75	1	0
4.97	3	2	1	1.5	2.083333	3.583333333	18	1
3.58	2.3	3	1	4.416667	1.333333	5.75	16	1
3.35	1.33	3	1	6	1.25	7.25	10	1
4.34	0.89	3	1	2	1.25	3.25	10	0
1.25	4.1	3	1	3.5	1.333333	2	5	0
4.78	1.45	3	1	3	1.5	4.5	7	0
4.66	3.8	3	1	3	1.366667	4.033333333	9	0
4.75	3.2	3	2	0.416667	1	4	6	0
4.77	3.3	2	2	6.25	1.333333	7.583333333	6	0
5.45	3.6	3	1	4	0.816667	6.233333333	3	0
3.62	1.8	3	1	2	0.5	2.5	8	0
3.67	2.1	3	1	2	2.633333	4.633333333	13	0
4.2	2.9	3	1	1.85	1.733333	3.583333333	5	0
4.03	1.31	3	1	1.5	2	3.5	20	0
4.16	3	3	1	1.5	1.25	2.75	4	0
4.82	3.3	3	4	0.833333	1.533333	3.7	27	0
2.71	1.6	3	1	1.7	0.8	2.5	6	0
5.65	4.3	3	1	3.25	1.333333	4.583333333	14	1
5.06	1.34	3	2	3	3	6	18	1
3.79	2.3	2	1	1.75	1.033333	1.833333333	12	0
3.82	2.4	3	4	3	0.933333	1.933333333	6	0
4.35	3.1	3	1	4.283333	1.216667	5.5	4	0
5.24	3.3	3	1	5.833333	2.5	8.333333333	11	0
7.03	1.67	3	1	2	1.5	3.5	8	0
6.4	3.5	3	2	1.75	2.383333	4.133333333	4	0
4.82	3.2	3	1	1.833333	1.666667	3.5	13	1
3.67	2.4	3	1	0.166667	2.25	2.416666667	14	0

0.73	1	3	1	3.033333	4	5	6	0
4.58	1.24	3	1	3	1.2	4.2	7	0
4.23	1.73	3	2	5	1	6	9	0
6	4.1	3	1	1.75	1	2	19	0
5.4	0.87	3	1	5	1.2	6.2	6	0
4.61	2.8	3	1	3	1.4	5.566666667	4	0
3.55	2.2	3	1	0.583333	2.25	2.75	4	0
3.46	4.6	3	1	6	0.75	6.75	16	0
5.09	2.3	3	1	2	1.666667	3.666666667	8	0

ID	DM_value	TC	OCSP	NIHSS_Base	mRs
10	4.7	4.23	1	10	1
15	4.7	4.58	1	21	1
24	4.9	4.59	1	7	1
27	6.4	6.58	1	9	1
37	5.8	5.93	1	7	1
40	5.5	5.54	1	9	1
49	5.3	4.78	1	7	1
81	4.9	3.95	1	5	1
83	6.3	3.67	1	7	1
84	7.9	5.23	1	9	1
102	4.7	3.89	1	11	1
112	5.8	4.7	1	17	1
153	6.1	3.53	1	12	1
154	10.6	4.22	1	7	1
155	7	4.85	1	11	1
163	4.8	4.71	1	8	1
168	3.6	4.61	1	18	1
170	6.3	3.59	1	9	1
174	5	3.78	1	8	1
252	7.4	3.59	1	16	1
253	5.5	4.84	1	10	1
275	6	4.74	1	9	1
42	6.1	4.65	1	10	1
56	5.3	4.94	1	10	1
87	8	7.39	1	13	1
91	9.7	4.66	1	12	1
108	5.4	4.95	1	10	1
126	4.5	2.69	1	9	1
136	4.7	4.68	1	16	1
152	5	5.2	1	11	1
165	14.1	5.12	1	21	1
188	4.5	4.37	1	12	1
218	13.4	5.36	1	13	1
243	7	4.5	1	21	1
247	5.6	3.61	1	11	1
267	6	2.67	1	17	1
280	5.3	4.31	1	11	1
304	5	5.45	1	18	1
306	12.8	4.39	1	20	1
315	4.5	3.24	1	8	1
45	5.3	4.09	1	8	1
82	4.1	3.62	1	21	1
134	6.1	4.52	1	16	1
164	6.7	4.18	1	14	1
173	4.4	3.62	1	22	1
195	4.5	6.1	1	12	1
223	8.1	5.57	1	15	1

231	5.5	5.77	1	19	1
285	4.5	4.81	1	17	1
292	4.2	1.25	1	12	1
301	4.4	6.15	1	14	1
92	5.3	4.52	1	22	1
232	4.8	4.94	1	20	1
248	5.2	4.77	1	22	1
6	4.3	3.98	1	7	0
30	4.4	5.1	1	6	0
36	4.9	3.6	1	7	0
58	5.3	4.24	1	10	0
64	4.5	5.94	1	7	0
70	3.8	4.65	1	6	0
78	6	5.96	1	9	0
85	3.9	7.03	1	5	0
103	11.5	5.07	1	10	0
109	4.2	4.34	1	5	0
114	3.7	4.23	1	7	0
121	6.4	4.71	1	10	0
132	4.4	4.29	1	7	0
133	5	4.89	1	6	0
137	4.7	0.56	1	6	0
151	4.6	4.9	1	7	0
187	5.3	5.48	1	4	0
191	4.5	2.59	1	7	0
192	5.1	4.76	1	6	0
196	4.8	3.42	1	4	0
198	4.7	2.55	1	10	0
208	5.7	6.46	1	8	0
230	4.5	4.53	1	7	0
233	5.8	4.66	1	6	0
234	6	5.41	1	8	0
241	9.2	4.34	1	10	0
245	4.9	4.04	1	13	0
259	5.3	5.28	1	5	0
269	6.2	4.13	1	6	0
272	15.3	3.55	1	9	0
283	3.9	6.4	1	6	0
294	9.4	4.58	1	6	0
296	5.9	5.86	1	5	0
312	9.5	4.02	1	11	0
314	4.1	3.67	1	9	0
0	3.7	2.84	1	5	0
3	4.7	4.26	1	5	0
5	5.5	6.33	1	5	0
13	5.5	3.59	1	5	0
16	4.8	2.7	1	7	0
18	6	4.95	1	11	0

19	6.2	5.05	1	6	0
20	5.9	3.77	1	6	0
21	5.2	3.65	1	6	0
25	5.3	3.46	1	6	0
26	6	3.29	1	7	0
32	5.1	5.25	1	6	0
33	7.7	2.75	1	7	0
35	7.1	3.81	1	6	0
41	4.6	3.16	1	6	0
44	4.3	4.21	1	7	0
46	11.7	5.36	1	5	0
50	4.2	5.48	1	11	0
51	4.7	5.27	1	4	0
52	7.6	5.43	1	4	0
54	4.8	4.67	1	5	0
55	5.3	7.49	1	4	0
57	5.8	5.77	1	4	0
59	6.4	5	1	12	0
60	5.1	3.48	1	4	0
61	5.5	5.25	1	4	0
62	6.1	2.38	1	7	0
66	4.5	5.27	1	8	0
71	4.7	2.36	1	12	0
73	5	2.76	1	4	0
86	4.5	4.89	1	9	0
89	4.4	4.02	1	5	0
96	7.9	4.77	1	4	0
101	6.3	4.89	1	4	0
107	4.6	3.95	1	8	0
111	7.3	5.19	1	6	0
113	5	3.14	1	4	0
115	4.9	5.02	1	8	0
116	6.5	5.69	1	13	0
118	5	4.56	1	6	0
119	4.5	4.53	1	11	0
120	7.6	4.34	1	8	0
123	9.2	3.66	1	11	0
127	5.2	3.8	1	7	0
129	13.8	4.29	1	7	0
130	4.2	3.35	1	7	0
131	6.1	3.52	1	8	0
139	5	4.63	1	5	0
143	8	4.04	1	7	0
148	0.9	5.09	1	7	0
150	4.7	5.47	1	12	0
157	5.6	6.69	1	8	0
160	5.5	4.27	1	6	0
167	4.8	5.65	1	5	0

169	6.4	5	1	6	0
171	5.3	4.62	1	7	0
176	4.4	4.7	1	7	0
180	5	4.77	1	15	0
193	4.9	3.81	1	5	0
194	4.4	3.97	1	7	0
197	6.4	4.88	1	12	0
199	4.9	5.14	1	11	0
200	4.23	4.97	1	4	0
202	6	3.89	1	12	0
203	4.9	3.42	1	4	0
205	4.1	4.03	1	6	0
209	4.8	4.51	1	6	0
210	5.4	4.57	1	4	0
211	4.6	3.63	1	9	0
216	4.1	4.16	1	4	0
217	5.8	4.34	1	8	0
224	4.4	4.49	1	5	0
225	5	3.71	1	4	0
227	4.3	5.21	1	8	0
235	3.9	4.35	1	20	0
240	7.3	1.92	1	6	0
242	5.1	3.93	1	6	0
249	5.1	4.61	1	8	0
256	4.9	4.42	1	4	0
258	4.8	4.39	1	5	0
261	4.8	3.91	1	9	0
263	5.8	4.51	1	4	0
265	4.8	5.35	1	5	0
268	4.7	3.65	1	6	0
270	8	5.57	1	5	0
273	4	3.79	1	4	0
282	4.3	3.36	1	6	0
284	3.6	3.55	1	7	0
286	5.4	4.69	1	5	0
287	4.7	2.65	1	4	0
289	5.11	2.68	1	4	0
290	6.1	4.4	1	12	0
291	5.6	5.02	1	4	0
293	5.6	3.74	1	5	0
297	4.8	3.79	1	5	0
299	6.3	4.21	1	5	0
305	4.1	4.2	1	19	0
308	4.7	3.44	1	5	0
11	4.2	4.75	2	10	1
23	8.2	4.63	2	6	1
31	5.1	4.26	2	14	1
65	5.3	3.66	2	6	1

88	15.1	4.42	2 7	1
95	5.4	4.45	2 18	1
98	5.5	4.41	2 7	1
117	4.9	3.05	2 7	1
124	5.4	4.58	2 13	1
142	5.3	5.27	2 5	1
184	7.6	3.91	2 10	1
204	4.5	5.22	2 9	1
250	7.9	4.91	2 8	1
251	5	3.99	2 7	1
277	5.1	4.2	2 8	1
278	10.63	3.82	2 7	1
281	5.4	6.4	2 8	1
300	5.4	5.54	2 12	1
302	4.7	4.34	2 9	1
310	4.8	4.43	2 14	1
313	6.3	4.05	2 7	1
75	4.8	3.55	2 14	1
162	3.8	3.67	2 12	1
166	5	4.53	2 5	1
229	6.4	4.16	2 8	1
255	4.6	4.44	2 10	1
17	4.2	3.58	2 13	1
38	4.4	4	2 10	1
221	4.8	4.46	2 17	1
125	5.6	4.9	2 13	1
48	7.1	3.89	2 16	1
1	4.7	4.55	2 8	0
28	4.1	2.71	2 16	0
29	4.8	4.41	2 9	0
43	4.6	5.56	2 11	0
72	4.6	5.06	2 6	0
79	6.1	4.79	2 5	0
106	8.6	5.52	2 11	0
128	14.1	5.25	2 9	0
135	4.6	5.38	2 5	0
141	7.1	4.51	2 9	0
144	4.3	4.34	2 15	0
156	4.2	4.66	2 10	0
172	6.3	4.44	2 6	0
175	13.4	5.27	2 11	0
177	11.8	4.99	2 4	0
178	4.9	4.37	2 10	0
183	7.8	5.88	2 4	0
186	8.3	3.93	2 6	0
206	5.4	5.4	2 4	0
222	5	3.9	2 11	0
236	5.5	7.26	2 7	0

239	8.1	4.12	2	6	0
246	10.4	3.38	2	7	0
260	5.7	4.77	2	7	0
264	4.7	4.32	2	5	0
271	7	3.95	2	7	0
276	10.6	4.86	2	5	0
303	6.63	4.43	2	7	0
2	4.6	4.47	2	4	0
4	5	4.64	2	5	0
7	5.3	4.96	2	5	0
8	9.5	6.84	2	8	0
9	4.4	5.36	2	8	0
12	4.4	3.56	2	11	0
14	5.7	4.02	2	5	0
22	5.5	4.45	2	12	0
34	7.8	6.56	2	6	0
39	5.5	6.25	2	7	0
47	7.1	4.11	2	12	0
53	8.2	3.91	2	14	0
63	4.5	5.1	2	4	0
67	6.5	5.07	2	7	0
68	5.5	4.24	2	4	0
69	5.2	4.24	2	6	0
74	7.2	5.32	2	6	0
76	4.8	5.71	2	6	0
77	5.5	3.73	2	7	0
80	5.3	3.28	2	7	0
90	4.6	3.02	2	6	0
93	6.2	4.92	2	6	0
94	4.7	5.12	2	8	0
97	5.1	4.44	2	7	0
99	6.4	5.62	2	7	0
100	6.9	4.8	2	5	0
104	4.8	5.79	2	6	0
105	20.6	3.37	2	5	0
110	5.5	4.41	2	7	0
122	6.5	3.39	2	6	0
138	5	3.91	2	4	0
140	4.4	5.32	2	6	0
145	7	5.07	2	4	0
146	7.3	3.37	2	7	0
147	5.8	6.34	2	4	0
149	6.1	5.03	2	6	0
158	6.7	4.97	2	9	0
159	5	3.85	2	10	0
161	4	5.65	2	6	0
179	5	4.13	2	6	0
181	3.7	6	2	7	0

182	8.7	5.6	2	4	0
185	5.1	7.13	2	11	0
189	5.9	5.09	2	4	0
190	6.4	5.95	2	13	0
201	9.5	4.51	2	4	0
207	5	5.4	2	6	0
212	6.2	8.51	2	4	0
213	5.5	5.24	2	6	0
214	8.1	3.59	2	12	0
215	4.8	4.11	2	6	0
219	3.8	4.58	2	5	0
220	6.8	5.73	2	6	0
226	4.7	3.89	2	7	0
228	5.3	2.47	2	6	0
237	5.6	3.04	2	9	0
238	4.8	3.59	2	6	0
244	5	3.49	2	5	0
254	4.4	5.14	2	5	0
257	4.8	3.38	2	11	0
262	7.1	3.95	2	9	0
266	5.5	4.53	2	8	0
274	7.5	4.8	2	6	0
279	6.3	5.08	2	7	0
288	12.8	6.25	2	6	0
295	4.6	2.71	2	7	0
298	5.4	5.78	2	7	0
307	4.7	4.19	2	8	0
309	4.5	3.84	2	4	0
311	4.7	3.27	2	19	0

ID	DM_value	TC	OCSP	NIHSS_Base	mRs
1	4.7	3.73	2	11	1
3	5.5	5.02	2	6	1
12	6.2	2.49	2	7	1
19	9.5	3.18	2	13	1
20	9.5	3.4	2	15	1
21	4.4	4.54	2	9	1
25	5	3.49	2	13	1
28	4.9	7.29	2	20	1
35	10	4.21	2	5	1
37	6.7	2.22	2	8	1
65	4.3	3.93	2	6	1
72	7	4.19	2	12	1
79	5.5	6.1	2	7	1
97	5.3	4.96	2	10	1
106	6.4	5.08	2	13	1
7	5.6	5.61	1	13	1
9	3.8	4.82	1	13	1
13	5.6	5.6	1	13	1
30	4.9	1.37	1	14	1
41	4.9	4.52	1	10	1
56	6.4	5.3	1	13	1
57	5.4	4.57	1	10	1
61	4.9	4.18	1	14	1
62	5.7	3.89	1	17	1
67	6.6	5.43	1	23	1
69	10.6	4.68	1	14	1
70	5.7	4.13	1	16	1
75	5.1	3.53	1	20	1
77	8.5	3.78	1	7	1
86	4.4	3.49	1	11	1
88	4.8	2.96	1	10	1
92	5.8	6.34	1	8	1
100	5.3	5.01	1	4	1
103	4.3	6.6	1	13	1
104	4.8	4.16	1	9	1
105	7	5.14	1	18	1
108	6.5	5.84	1	10	1
111	3.46	3.46	1	16	1
0	5.7	3.85	2	6	0
4	6.3	3.27	2	8	0
5	4.4	4.9	2	4	0
8	5.2	4.7	2	6	0
10	5	5.14	2	4	0
11	4.3	4.79	2	6	0
14	6.7	4.49	2	7	0
16	5.4	3.27	2	8	0
18	6.7	4.49	2	8	0

22	4.6	5.78	2	5	0
24	4.4	5.46	2	8	0
27	5.6	5.18	2	14	0
36	11.5	4.36	2	5	0
39	5.4	3.51	2	8	0
40	4.9	2.91	2	8	0
50	5.6	3.07	2	5	0
58	7.7	4.66	2	9	0
59	5.6	3.07	2	5	0
60	5.2	4.58	2	4	0
64	5.2	4.48	2	5	0
66	5.5	3.87	2	8	0
73	4.6	5.26	2	7	0
74	6.5	4.77	2	13	0
81	5.4	4.53	2	8	0
83	4.7	3.3	2	8	0
84	4.7	5.86	2	5	0
90	6	5.26	2	6	0
95	5	3.8	2	6	0
101	6.2	5.41	2	9	0
109	5.2	4.7	2	4	0
112	5.2	4.7	2	4	0
2	5.8	4.68	1	6	0
6	6.7	1.32	1	8	0
15	6.8	4.46	1	6	0
17	5	4.01	1	7	0
23	7.8	4.28	1	11	0
26	7.8	4.2	1	11	0
29	4.3	3.44	1	5	0
31	8.3	5.35	1	6	0
32	9.1	4.27	1	9	0
33	5	7.53	1	5	0
34	5	7.6	1	5	0
38	5.8	4.55	1	6	0
42	4.7	5.47	1	10	0
43	9.9	4.58	1	6	0
44	5.2	4.79	1	6	0
45	5.4	5.35	1	4	0
46	4.2	4.78	1	7	0
47	10.3	3.52	1	7	0
48	6.6	5.84	1	8	0
49	5.1	3.64	1	6	0
51	6	4.96	1	12	0
52	5.3	2.64	1	8	0
53	4.3	5.62	1	9	0
54	6.4	4.16	1	4	0
55	4.3	5.62	1	9	0
63	4.6	3.39	1	5	0

68	5.3	3.86	1	5	0
71	5.3	3.83	1	6	0
76	7	5.9	1	7	0
78	4.3	4.95	1	7	0
80	4.3	4.95	1	7	0
82	4.3	4.95	1	8	0
85	4.6	4.75	1	6	0
87	4.9	4.28	1	6	0
89	6.1	4.16	1	13	0
91	11	6.99	1	18	0
93	5.4	3.52	1	8	0
94	5.3	5.13	1	9	0
96	7.8	4.3	1	6	0
98	10.8	5.09	1	4	0
99	4.7	4.12	1	4	0
102	5.2	5.24	1	6	0
107	4.5	4.12	1	5	0
110	5.8	4.8	1	5	0
113	5.2	5.46	1	5	0
